# Autonomic dysregulation and self‐injurious thoughts and behaviours in children and young people: A systematic review and meta‐analysis

**DOI:** 10.1002/jcv2.12148

**Published:** 2023-03-23

**Authors:** Alessio Bellato, Muskaan Aleeza Admani, Camila Deak, Luis Carlos Farhat, Maria Carolina Fontana Antunes de Oliveira, Rebeca Vasconcelos, Margherita Malanchini, Elizabeth Shephard, Giorgia Michelini

**Affiliations:** ^1^ School of Psychology University of Nottingham Semenyih Malaysia; ^2^ Department of Biological and Experimental Psychology School of Biological and Behavioural Sciences Queen Mary University of London London UK; ^3^ Department of Psychiatry Faculdade de Medicina Universidade de São Paulo Sao Paulo Brazil; ^4^ Department of Psychology Health Sciences Center Universidade de Fortaleza Sao Paulo Brazil; ^5^ Institute of Psychiatry, Psychology & Neuroscience (IoPPN) King's College London London UK; ^6^ Semel Institute for Neuroscience and Human Behavior University of California Los Angeles Los Angeles California USA

**Keywords:** arousal, autonomic, electrodermal, heart rate variability, self‐harm, suicide

## Abstract

**Background:**

Self‐injurious thoughts and behaviours (SITBs) have been associated with dysfunction of the Autonomic Nervous System (ANS) in children and young people, suggesting that objective ANS measures may aid assessment of suicide risk, but a systematic synthesis of this literature is currently lacking.

**Methods:**

Following a pre‐registered protocol (PROSPERO CRD42022327605), we conducted a systematic search of PubMed, Medline, Embase, PsycINFO, and Web of Science, for empirical studies published until 10th May 2022 that compared indices of ANS functioning in individuals aged 0–25 years with versus without SITBs, or reported continuous associations between ANS measures and SITBs. Study quality was assessed with the Newcastle‐Ottawa Scales. Pooled effect sizes (Hedge's g) were estimated with random‐effects meta‐analytic models.

**Results:**

Twenty studies (1979 participants) were included in our systematic review, with 16 included in meta‐analyses. Results suggested that SITBs were associated with altered cardiac indices of arousal (*g* = −0.328, *p* < 0.001), which was driven by lower heart rate variability in individuals with SITBs (*g* = −0.375, *p* = 0.025). Overall results for electrodermal activity were not significant (*g* = 0.026, *p* = 0.857), but subgroup analyses showed increased activity in studies of individuals who engaged specifically in non‐suicidal self‐harm (*g* = 0.249, *p* = 0.014) but decreased activity in the remaining studies (*g* = −0.567, *p* = 0.004).

**Conclusions:**

Our systematic review and meta‐analysis found evidence of reduced parasympathetic regulation as well as more tentative evidence of altered electrodermal activity in children and young people displaying SITBs. Future longitudinal studies should test the clinical utility of these markers for detecting and monitoring suicide risk.

## Graphical abstract

Self‐injurious thoughts and behaviours: Meta‐analysis of the association between autonomic dysregulation and suicide risk in children and young people: we found that reduced cardiac regulation and altered electrodermal activity characterise children and young people displaying self‐injurious thoughts and behaviours (SITBs). Future large longitudinal studies should further clarify how these associations unfold over time, to inform tools for early identification and prevention of SITBs in children and young people.

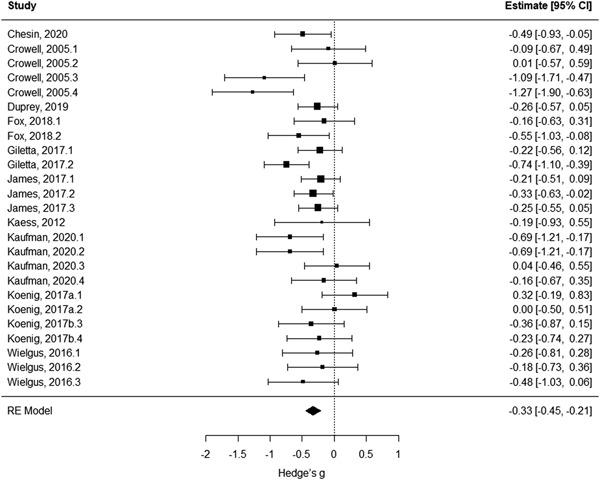

**Key points**

- Previous studies have associated self‐injurious thoughts and behaviours (SITBs) with autonomic dysregulation (e.g., reduced electrodermal activity and heart rate variability).
- To further clarify this association, a systematic review and meta‐analysis was conducted; we found that reduced cardiac regulation and altered electrodermal activity characterise children and young people displaying SITBs.
- Future studies, especially using longitudinal designs and larger and heterogeneous samples, are needed to further clarify the specific associations between autonomic dysregulation and SITBs.
- This, in turn, could prove helpful for developing tools for early identification of children and young people at highest risk of engaging in self‐harming behaviours, with the ultimate goal of reducing the number of children and young people dying by suicide.

## INTRODUCTION

Children and young people are at high risk for self‐injurious thoughts and behaviours (SITBs), which include suicidal ideation (i.e., any thought, contemplation, idea or plans of committing suicide in the short‐ or long‐term period), suicidal self‐harm (i.e., self‐injurious behaviours implemented to take one's own life; namely suicide attempts) and non‐suicidal self‐harm (i.e., intentional alteration or destruction of body tissue with the aim of self‐injury but without suicidal intent; Klonsky, 2007). Global estimates indicate that up to one in four children and young people experience suicidal ideation and/or engage in self‐harm (Biswas et al., 2020; Lawrence et al., 2021; Lim et al., 2019; Liu et al., 2022; Mercado et al., 2017). Besides being distressing and impairing, SITBs are the strongest predictors of future suicide, one of the leading causes of death amongst children and young people (Witt et al., 2021). These alarming figures highlight the necessity of continuous efforts to find effective ways to identify those at highest suicide risk.

SITBs often arise in association with a range of mental health conditions, such as mood disorders, personality disorders, and neurodevelopmental conditions (Liu et al., 2022; O’Connor, 2021; Septier et al., 2019), but may also occur in individuals at risk for or with subthreshold presentations of these diagnoses, especially in childhood and adolescence (O’Connor, 2021). Previous research has linked SITBs with several clinical features and risk factors that cut across diagnostic boundaries (i.e., transdiagnostic), including emotion dysregulation and related risk factors (e.g., adverse childhood experiences; Cipriano et al., 2017; Li et al., 2022). This is consistent with the hypothesis that SITBs (especially, non‐suicidal self‐harm) may have a self‐regulatory function, such that people may engage in these behaviours to regulate emotion and compensate for a dysregulated Autonomic Nervous System (ANS; Glenn et al., 2018; van Hoorn, 2020). The ANS is a core component of the peripheral nervous system that controls key physiological functions, including heart rate (number of heart beats per minute), perspiration (production of fluids secreted by the skin's sweat glands), and gastro‐intestinal functions. Importantly, a significant association between autonomic dysregulation and emotional dysregulation in children and young people (especially in relation to cardiac measures obtained at rest) has been recently demonstrated (Bellato et al., 2023).

Previous studies have associated suicidality in young people with several indices of autonomic dysregulation, such as reduced electrodermal activity and lower heart rate variability (HRV; see systematic reviews by Sarchiapone et al., 2018; Kang et al., 2020). Prefrontal and limbic brain regions, which are involved in autonomic regulation in response to negative events, are also less active in people experiencing SITBs (Westlund Schreiner et al., 2015; Yoon et al., 2022). However, inconsistent findings have also emerged in the literature (e.g., Aldrich et al., 2018; Koenig et al., 2018), likely owing to the use of small samples and methodological differences. Moreover, previously published systematic reviews did not consider non‐suicidal self‐harm and did not include formal meta‐analyses.

We thus carried out a systematic review and meta‐analysis to quantify the strength and consistency of the association between markers of autonomic functioning and SITBs in children and young people. Given the relationship between different forms of SITBs, we investigated them together but also separately, to elucidate possible differences in their associations with autonomic dysregulation. Considering the importance of adopting a transdiagnostic approach for investigating SITBs, we did not restrict our focus to samples of individuals with specific psychiatric conditions. We included young people up to 25 years to reflect recent developmental research expanding the definition of adolescence to also include young adulthood (Jaworska & MacQueen, 2015), and because SITBs are similarly prevalent in young people aged 12–18 years and 19–25 years (Biswas et al., 2020; Lim et al., 2019; Liu et al., 2022; Mercado et al., 2017).

## MATERIALS AND METHODS

### Search strategy and selection criteria

We followed the 2020 PRISMA guidelines (Page et al., 2021; Checklist is reported in Appendix S1) and pre‐registered our study on PROSPERO (CRD42022327605). We searched PubMed, Medline, Embase, PsycINFO, Web of Science for suitable studies in line with our main research question, summarised in the following PECO.

- Participants: Children and young people up to 25 years of age.
- Exposure: Presence of SITBs, ascertained via questionnaire or clinical interview.
- Comparator: Absence of SITBs.
- Outcome(s): Markers of autonomic functioning (e.g., heart rate, electrodermal activity or pupillometry).

The search included full journal articles or published abstracts from the beginning of time until 10th May 2022. The search strategy (Appendix S2) included terms associated with (a) Autonomic arousal and (b) Suicidal ideation, suicidal self‐harm and non‐suicidal self‐harm. Cross‐sectional and cohort studies including at least one measure of autonomic functioning (e.g., heart rate, electrodermal activity or pupillometry) in association with SITBs (ascertained via questionnaire or clinical interview) in individuals aged 0–25 years were eligible for inclusion. In relation to the latter, we only included studies that involved participants whose age ranged between 0 and 25 years, regardless of the reported mean age of the sample, and thus excluded studies that included participants older than 25 years. Previous systematic or narrative reviews' reference lists were searched to identify further studies meeting inclusion criteria.

### Data selection, extraction and quality rating

Titles and abstracts of retrieved studies were independently screened by three authors (AB, GM and ES) to identify those meeting inclusion criteria. Full texts of potentially eligible studies were also assessed by the same authors. Data extraction and assessment of data quality was performed by six authors (AB, MA, CD, LF, MF, RV). Disagreements were settled through discussion. Study quality and risk of bias was assessed with the Newcastle‐Ottawa Scale (NOS; Wells et al., 2014) for cross‐sectional and cohort studies. The NOS is a widely used tool that provides an evaluation of the overall quality of non‐randomised studies (either cross‐sectional or cohort), based on three main criteria: (a) selection of groups, (b) comparability of the study groups and (c) ascertainment of exposure (for case‐control studies) or study outcomes (for cohort studies). Unresolved classification of studies was arbitrated by the first author.

### Data synthesis and analysis

Studies were included in the meta‐analysis if they provided raw data for effect sizes to be computed (i.e., mean and standard deviation or standard error, for each group of participants with and without SITBs; correlation coefficients for the association between indices of autonomic arousal and severity of SITBs; or full statistical analyses of independent samples *t*‐test investigating differences in indices of autonomic arousal for participants with and without SITBs). For case‐control studies, we calculated Hedge's g as the standardised mean difference, while for cohort studies we converted correlation coefficients in Hedge's g. Random‐effects meta‐analyses estimated the pooled effect size using *metafor* (Viechtbauer, 2010) in *R 4.1.2* (R Core Team, 2020), whenever at least two studies reported on the same ANS measure. Effect sizes were nested within studies in multilevel models for those that reported multiple effect sizes for the same measure domain (cardiac or electrodermal) to account for data non‐independence, using the Restricted Maximum‐Likelihood estimator. Cross‐study heterogeneity was tested with Cochran *Q*. Funnel plots were inspected, and the rank correlation test was used to examine funnel plot asymmetry (which may have arisen from publication bias), followed by trim and fill analyses. Based on the retrieved studies – presented in the ‘Results’ section below – we tested two meta‐analytic models examining the association of SITBs with (1) cardiac measures of autonomic functioning (i.e., heart rate (HR) and heart rate variability (HRV); pre‐ejection period (PEP), respiratory sinus arrhythmia (RSA) and root mean square of successive differences between heartbeats (RMSSD); see Appendix S6 for more information) and (2) electrodermal measures (i.e., skin conductance level (SCL) and responses/reactivity (SCRs); see Appendix S7 for more information). Sub‐group analyses were conducted to investigate if meta‐analytic results differed between (a) studies reporting on children/adolescents versus young adults, (b) studies reporting measures obtained during resting‐state versus task, and (c) studies involving people with different types of SITB and (d) type of cardiac measure, because different cardiac metrics are thought to reflect different autonomic mechanisms, for example, sympathetic or parasympathetic. Results of sub‐group analyses (which were carried out in *metafor*) indicated whether the pooled effect size differed between subgroups. If the analysis reported a *p*‐value <0.05, it was suggested that subgroups significantly differ in the pooled effect size; when this was the case, we followed‐up the results by investigating the pooled effect size for each subgroup.

Further information is available in Appendix S2 and S3. Raw data are available at the link indicated in section ‘Data availability statement’. A qualitative synthesis is provided for studies with insufficient data for the meta‐analysis.

## RESULTS

### Characteristics of studies included in the review

**FIGURE 1:**
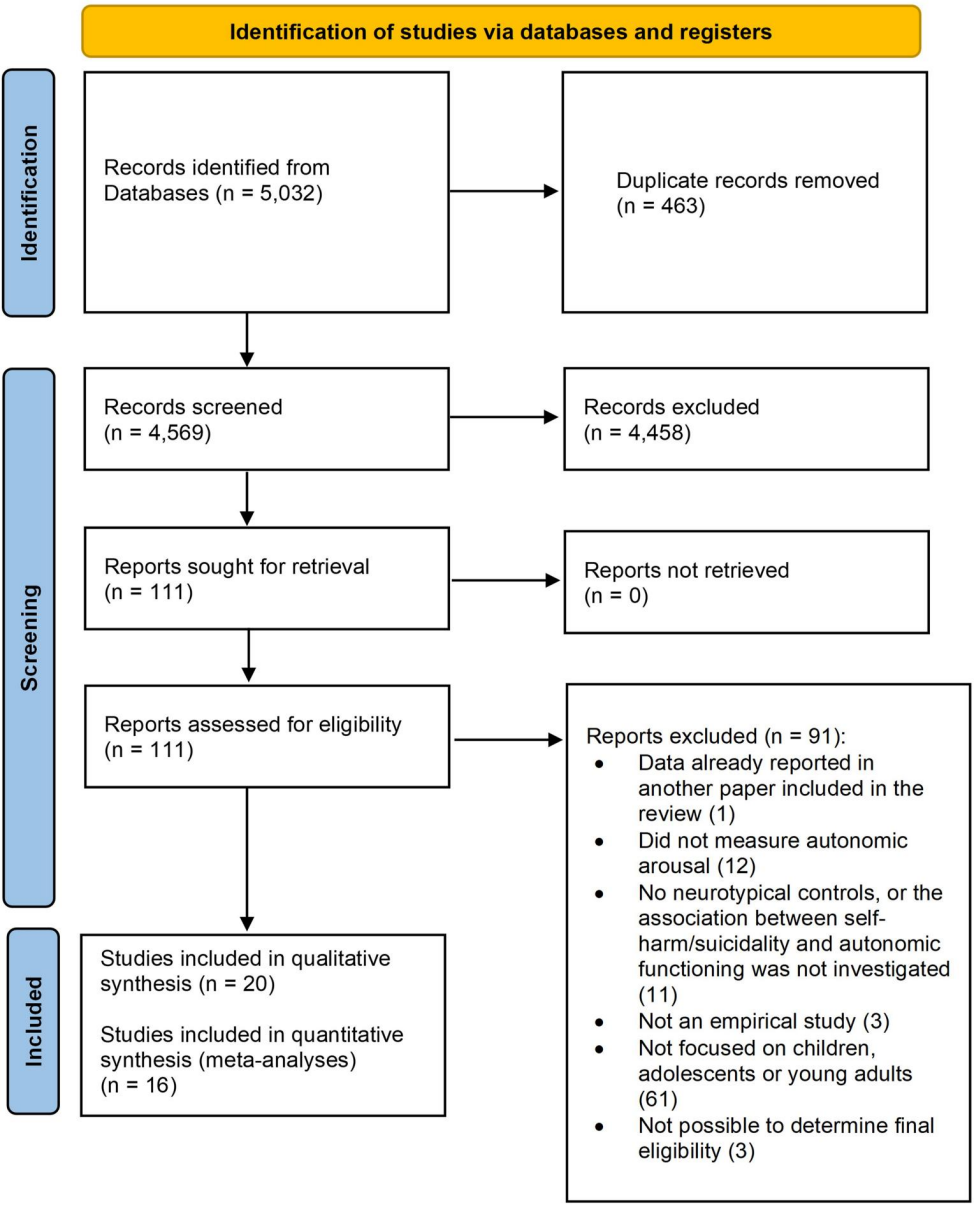
PRISMA flowchart.

Our initial search retrieved 5032 references, of which 463 were duplicates and 4458 were deemed ineligible based on title/abstract. After full‐text screening of the remaining 111 references, 20 met inclusion criteria and were included in the review (total *N* = 1979 children and young people; 39.4% reporting SITBs; 76% females) (Figure 1, Table 1; see Appendix S4 for list of excluded studies). Fifteen were case‐control studies and five were cohort studies (two community cohorts and three clinical cohorts). Fourteen studies included children and/or adolescents, while six recruited young adults (18–25 years). Four studies were on suicidal ideation, one on suicidal self‐harm, two on suicidal ideation/self‐harm, eight on non‐suicidal self‐harm, and five on SITBs more broadly (i.e., suicidal ideation and/or suicidal/non‐suicidal self‐harm). Thirteen studies focussed on cardiac measures as indices of autonomic functioning, six on electrodermal measures, and one on both. Three studies reported baseline/resting‐state autonomic measures, five reported task‐related autonomic measures, and 11 reported both. Twelve studies examined SITBs in other clinical conditions; the most common were major depression disorder, social anxiety disorders and borderline personality disorder. Of the 20 eligible studies, 16 were included in the meta‐analyses, and four in the narrative review (since effect sizes could not be computed for Crowell et al., 2012; Sheridan et al., 2021 ; Yang et al., 2019; and for Koenig et al., 2018, relevant data was already reported in Koenig, Rinnewitz, Parzer, et al., 2017). Out of 14 cross‐sectional studies, only one was of good quality, seven of fair quality, and six of poor quality, while one cohort study was of good quality and five of poor quality (Appendix S5; Tables S1 and S2).

**TABLE 1:** Characteristics of studies investigating autonomic functioning in relation to SITBs.

Study	Included in meta‐analysis	Sample characteristics	Ethnicity	Other diagnoses	SITB domain	Autonomic measure(s)	Experimental task/activity	SITB assessment(s)	Summary of findings
Aldrich et al. (2018)	Electrodermal	SITB group: *n* = 16 (mean age: 12.64 years; 62.5% F); Control group: *n* = 96 (mean age: 12.89 years; 54.3% F)	Not reported	None reported	All SITBs	Electrodermal: SCR	Baseline/Resting‐state, Anagram solving task	Youth Self‐Report, Children's Depression Inventory‐2, Kiddie Schedule of Affective disorders and Schizophrenia	No group differences in SCR at rest and during task
Chesin et al. (2020)	Cardiac	SITB group: *n* = 9 (mean age: 20 years; 72% F); Control group: *n* = 72 (mean age: 20 years; 72% F)	Both groups: 38% White; 0% Asian; 17% Black; 45% other.	None reported	Suicidal ideation	Cardiac: HRV	Arousal inducing tasks: Stroop task, Cyberball task	Beck Scale for suicide ideation	Higher suicidal ideation associated with reduced autonomic arousal regulation (HRV) during tasks compared to baseline.
Crowell et al. (2005)	Cardiac, electrodermal	SITB group: *n* = 23 (mean age: 15.3 years; 100% F); Control group: *n* = 23 (mean age: 15.3 years; 100% F)	Both groups: 81% White; 0% Asian; 4% Black; 15% other.	Dysthymia, generalised anxiety, social phobia, bipolar disorder	All SITBs	Electrodermal/Cardiac: SCR, PEP, RSA	Baseline/Resting‐state, Sadness‐evoking video and Recovery	Lifetime Parasuicide Count/Lifetime Suicide Attempt and Self‐injury interview	Reduced RSA at rest and during recovery from videos in SITB group versus controls. RSA decreased over time in SITB group, but not in controls. No group differences during any conditions.
Crowell et al. (2012)	Qualitative synthesis only	SITB group: *n* = 27 (mean age: 16.3 years; 100% F); Control group: *n* = 24 (mean age: 16.1 years; 100% F)	Both groups: 83.3% White; 1.2% Asian; 4.8% Black; 10.7% other.	Borderline personality disorder	All SITBs	Electrodermal: SCR	Baseline/Resting‐state, Sadness‐evoking video	Lifetime Parasuicide Count; Lifetime Suicide Attempt and Self‐injury interview	Reduced skin conductance at baseline in SITB group versus controls. No differences during video.
Duprey et al. (2019)	Cardiac	Community sample: *n* = 163 (mean age: 21.17 years; 55.8% F)	62.2% White; 0% Asian; 33.1% Black; 4.7% other.	None reported	Suicidal ideation	Cardiac: HRV	Baseline/Resting‐state	Suicidal Ideation Scale	No association between suicidal ideation and resting HRV.
Fox et al. (2018)	Cardiac	Community sample: *n* = 70 (mean age: 19.25 years; 94% F)	71.4% White; 11.4% Asian; 7.1% Black; 10% other.	None reported	Non‐suicidal self‐harm	Cardiac: RSA	Baseline/Resting‐state, Stress task (preparation of public speech), Recovery	Inventory for Statements about Self‐injury	Non‐suicidal self‐harm engagement in the past year associated with lower RSA during task, but not with resting RSA.
Giletta et al. (2017)	Cardiac	SITB (at‐risk) sample: *n* = 132 (mean age: 14.59 years; 100% F)	66.7% White; % Asian; 24.2% Black; 9.1% other.	ADHD, conduct disorder, anxiety disorder, major depressive disorder	Suicidal ideation	Cardiac: RSA	Baseline/Resting‐state, Modified Trier social stress test	Self‐Injurious Thoughts and Behaviours interview	Increased suicide ideation associated with reduced RSA during a stressor task, but not with resting RSA.
James et al. (2017)	Cardiac	SITB group: *n* = 51 (mean age: 8.91 years; 44.2% F); control group: *n* = 242 (mean age: 9.54 years; 51.9% F)	SITB group: 82.7% White; % Asian; % Black; 17.3% other. Control group: 70.1% White; % Asian; % Black; 29.9% other.	None reported	Suicidal ideation	Cardiac: RSA	Baseline/Resting‐state, Vacation‐planning task with parents, discussion about issues with parents	Schedule for Affective disorders and Schizophrenia, Children's Depression Inventory	SITB group displayed lower RSA during a stressor task than controls. No differences at rest.
Kaess et al. (2012)	Cardiac	SITB group: *n* = 14 (mean age: 16.6 years; 100% F); control group: *n* = 14 (mean age: 16.3 years; 100% F)	Not reported	Borderline personality disorder, major depressive disorder, anxiety disorder, post‐traumatic stress disorder	Non‐suicidal self‐harm	Cardiac: HR reactivity	Trier Social Stress Test	Functional assessment of self‐mutilation	No group differences on HR reactivity in response to stress (task vs. baseline).
Kaufman et al. (2020)	Cardiac	60 adolescents. SITB group: *n* = 30 (mean age: 15.47 years; 100% F); control group: *n* = 30 (mean age: 14.77 years; 100% F)	Both groups: 91.67% White; 2.5% Asian; 1% Black; 5% other.	None reported	All SITBs	Cardiac: RSA	Baseline/Resting‐state, social interaction with mother, discussion with mother designed to provoke emotional arousal and disagreement, discussion with mother on conflict‐provoking topics	Lifetime Suicide Attempt and Self‐injury interview	SITB group displayed reduced RSA at rest and during a social interaction with their mother versus control group. No differences during other tasks.
Koenig et al. (2017a)	Cardiac	SITB group: *n* = 30 (mean age: 15.27 years; 100% F); control group: *n* = 30 (mean age: 15.27 years; 100% F)	Not reported	Borderline personality disorder	Non‐suicidal self‐harm	Cardiac: HR, RMSSD	Baseline/Resting‐state	Self‐Injurious Thoughts and Behaviours interview	No group differences on any measure.
Koenig, Rinnewitz,Warth, et al. (2017)	Cardiac	SITB group: *n* = 30 (mean age: 15.27 years; 100% F); control group: *n* = 30 (mean age: 15.27 years; 100% F)	Not reported	Borderline personality disorder, major depression disorder, social anxiety disorder	Non‐suicidal self‐harm	Cardiac: HR, RMSSD, SBP, DBP	Cold Pressor Test	Self‐Injurious Thoughts and Behaviours interview	No group differences on any measure.
Koenig et al. (2018)	Qualitative synthesis only	SITB sample: *n* = 17 (mean age: 15.24 years; 100% F)	Not reported	Borderline personality disorder	Non‐suicidal self‐harm	Cardiac: HR, RMSSD	Baseline/Resting‐state	Self‐Injurious Thoughts and Behaviours interview	Non‐significant correlation between non‐suicidal self‐harm and resting HR or RMSSD.
Nock and Mendes (2008)	Electrodermal	SITB group: *n* = 62 (mean age: 17.4 years; 79.7% F); control group: *n* = 30 (mean age: 16.7 years; 73.3% F)	SITB group: 75% White; 4.7% Asian; 3.1% Black; 0% other. Control group: 70% White; 6.7% Asian; 3.3% Black; 3.3% other.	Anxiety disorder, major depressive disorder, alcohol and substance use disorder, impulse‐control disorder, eating disorder	Non‐suicidal self‐harm	Electrodermal: SCR	Distress Tolerance Test based on Wisconsin Card Sorting Test	Self‐Injurious Thoughts and Behaviours interview	SITB group displayed increased skin conductance reactivity versus controls, especially when informed about their incorrect answers.
Sheridan et al. (2021)	Qualitative synthesis only	SITB sample: *n* = 51 (mean age: 16.70 years; 72.6% F)	Not reported	None reported	Suicidality (not specified if ideation or behaviours)	Cardiac: HRV	Continuous measurement (24h)	Columbia Suicide Severity Scale	A decrease in suicidality severity over time associated with increased HRV at rest during night‐time, but not during the day.
Tatnell et al. (2018)	Electrodermal	SITB group: *n* = 25 (mean age: 20.05 years; 66.7% F); control group: *n* = 53 (mean age: 20.05 years; 66.7% F)	Not reported	Anxiety disorder, obsessive compulsive disorder, major depressive disorder, borderline personality disorder, bipolar disorder	Non‐suicidal self‐harm	Electrodermal: SCL, SCR	Baseline/Resting‐state, Trier Social Stress Test, exposure to emotion‐inducing pictures	Inventory of Statements about Self‐Injury	No group differences in skin conductance reactivity during all conditions.
Tuna and Gencoz (2021)	Electrodermal	SITB group: *n* = 34 (mean age: 21.07 years; 60% F); control group: *n* = 36 (mean age: 21.07 years; 60% F)	Not reported	None reported	Non‐suicidal self‐harm	Electrodermal: SCL, SCR	Baseline/Resting‐state, Cold Pressor Test	Inventory of Statements about Self‐Injury	No group differences in skin conductance during all conditions.
Wang et al. (2021)	Electrodermal	SITB group: *n* = 36 (mean age: 19.485 years; 42.125% F); control group: *n* = 19 (mean age: 20.84 years; 52.63% F)	Not reported	Major depressive disorder, social phobia, generalised anxiety disorder	Suicide ideation/attempt	Electrodermal: SCR	Iowa Gambling Task	Mini International Neuropsychiatric Inventory, Beck Scale for Suicide Ideation, Beck Depression Inventory, State Anxiety Inventory	No group differences in skin conductance reactivity.
Wielgus et al. (2016)	Cardiac	SITB group: *n* = 15 (mean age: 12.46 years; 60% F); control group: *n* = 93 (mean age: 12.86 years; 50% F)	Both groups: 71.3% White; 7.4% Asian; 0.9% Black; 20.4% other.	Major depressive disorder	All SITBs	Cardiac: RSA	Baseline/Resting‐state, Cognitive Stressor Task and Recovery	Children's Depression Inventory, Youth Self Report, Child Behaviour Checklist, Schedule of Affective Disorders and Schizophrenia for School‐age Children	No group differences in RSA during all conditions.
Yang et al. (2019)	Qualitative synthesis only	SITB group: *n* = 177 (mean age: 17.1 years; 35% F); control group: *n* = 175 (mean age: 16.2 years; 34.9% F)	Not reported	Major depressive disorder, anxiety disorder	Suicidal thoughts and behaviours	Cardiac: RSA, PEP	Baseline/Resting‐state, Sad film clip, Unsolvable puzzle task	Interview Schedule for Children and Adolescents	No group differences on RSA or PEP during all conditions. RSA decreased (from baseline) in controls during the unresolved puzzles task, but not in SITB group.

Abbreviations: DBP, Diastolic Blood Pressure; HR, Heart Rate; HRV, Heart Rate Variability; PEP, Pre‐Ejection Period; RMSSD, Root Mean Square of Successive Differences; RSA, Respiratory Sinus Arrhythmia; SBP, Systolic Blood Pressure; SCL, Skin Conductance Level; SCR, Skin Conductance Response; SITB, Self‐Injurious Thought and Behaviour.

### Cardiac measures

**FIGURE 2:**
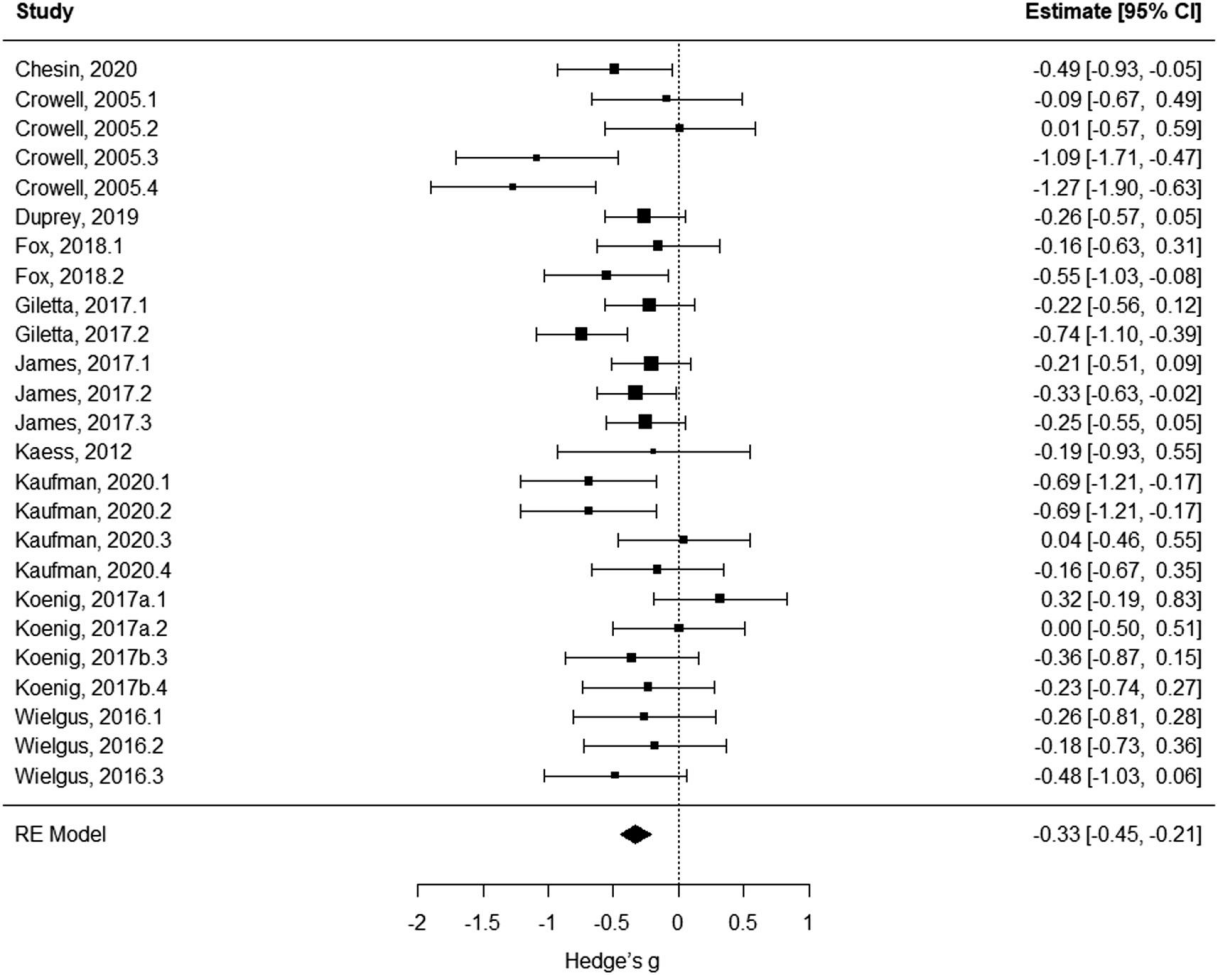
Forest plot of effect sizes for studies investigating cardiac measures. CI, confidence interval; RE, random effect.

Of 14 studies reporting cardiac measures, 10 (25 effects) were included in the meta‐analysis. We found a statistically significant negative association between cardiac measures and SITBs (Hedge's *g* = −0.328, SE = 0.058, 95% CI = [−0.447; −0.209], *t* = −5.667, *p* < 0.001; Figure 2; Table S3). Specifically, in individuals reporting SITBs we found cardiac markers of reduced autonomic arousal and arousal regulation. Cross‐study heterogeneity was significant (*Q* = 39.638; *p* = 0.023), while funnel plot asymmetry was not detected (Kendall's tau = −0.1510, *p* = 0.2927; Figure S1).

Subgroup analyses found no differences in results between studies reporting on different experimental conditions (baseline/resting‐state vs. task: *F*_1,23_ = 1.777, *p* = 0.196), developmental stage (children/adolescents vs. young adults: *F*_1,23_ = 0.011, *p* = 0.919), or SITB domain (suicidal ideation vs. suicidal self‐harm vs. non‐suicidal self‐harm: *F*_2,22_ = 1.293, *p* = 0.295). However, we found a significant effect of the type of cardiac measure (F_1,23_ = 5.761, *p* = 0.025), as SITBs were significantly associated with reduced parasympathetically‐mediated HRV (Hedge's *g* = −0.375, SE = 0.156, 95% CI = [−0.697; −0.052], *t* = −2.400, *p* = 0.025), but not with sympathetically‐mediated measures (e.g., PEP, HR; Hedge's *g* = −0.008, SE = 0.146, 95% CI = [−0.309; 0.294], *t* = −0.051, *p* = 0.960).

Consistent with these findings, one of the three studies included in the qualitative synthesis found that adolescents who displayed a decrease in suicidality over time showed an increase in HRV at rest during night‐time, but not during the day (Sheridan et al., 2021), whereas the other study found no group differences on RSA or PEP (Yang et al., 2019). Koenig et al. (2018) reported results of a 1‐year follow‐up assessment of a sample of adolescent girls, for which they reported baseline data in Koenig, Rinnewitz, Parzer, et al. (2017). They found that, 1 year after the first assessment, non‐suicidal self‐harm was reduced but this was not accompanied by any change in resting‐state vagally‐mediated HRV.

### Electrodermal measures

**FIGURE 3:**
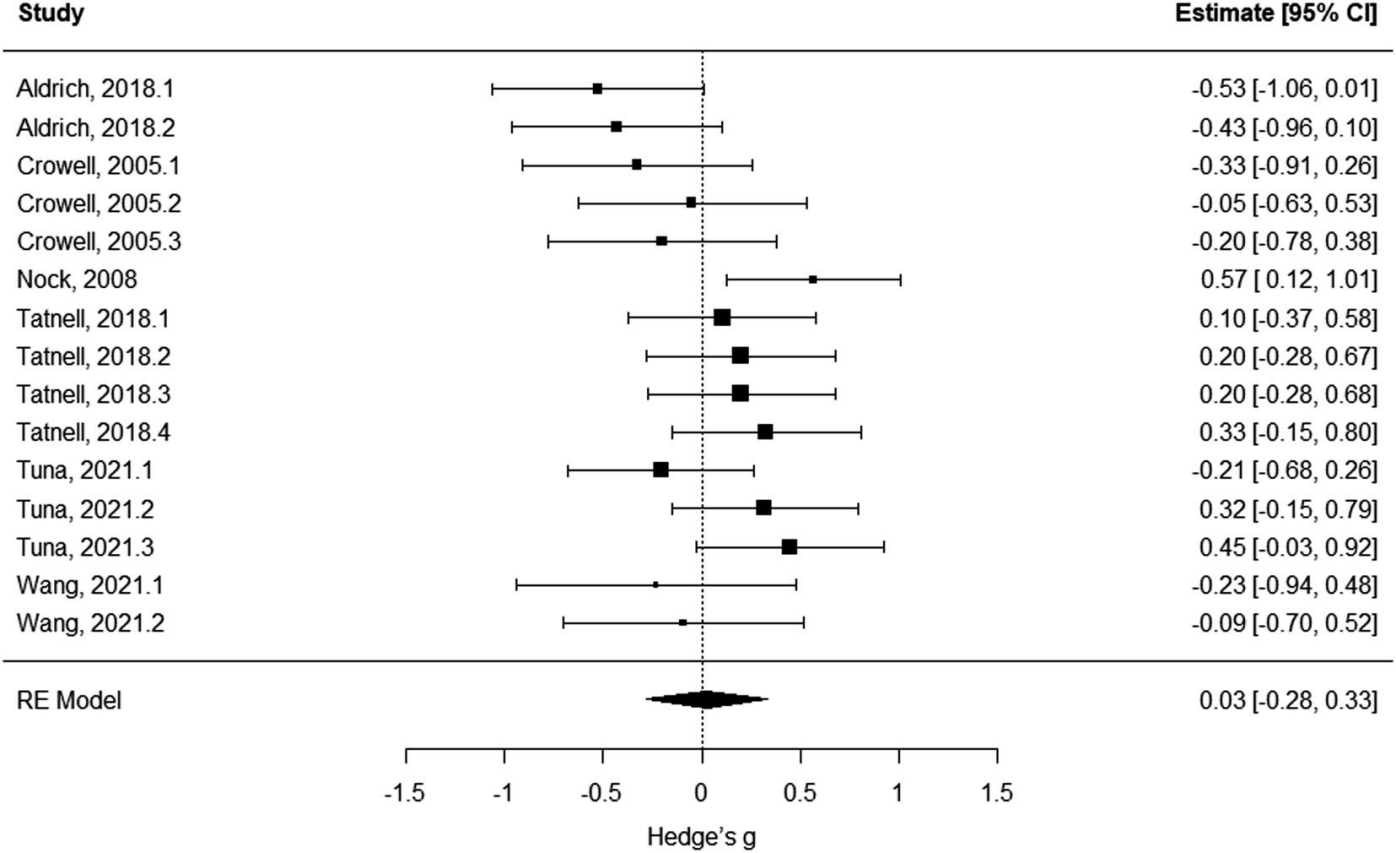
Forest plot of effect sizes for studies investigating electrodermal activity/reactivity. CI, confidence interval; RE, random effect.

Out of seven studies examining the association between electrodermal activity and SITBs, six (15 effects) provided sufficient information for the meta‐analysis. We did not find a statistically significant association between SITBs and electrodermal activity (Hedge's *g* = 0.026, SE = 0.142, 95% CI = [−0.279; 0.331], *t* = 0.184, *p* = 0.857; Figure 3; Table S4). Cross‐study heterogeneity was not significant (*Q* = 23.221; *p* = 0.057), while funnel plot asymmetry was detected (Kendall's tau = −0.402, *p* = 0.037; Figure S2). Two trim and fill sensitivity analyses (randomly selecting only one effect size for studies reporting more than one) showed that no study was missing due to publication bias.

Subgroup analyses did not find any changes in results between studies reporting on different type of electrodermal measure/experimental condition (skin conductance level during baseline/resting‐state vs. skin conductance response during task: *F*_1,13_ = 4.007, *p* = 0.067), or developmental stage (children/adolescents vs. young adults: *F*_1,13_ = 0.256, *p* = 0.621). There was a significant effect of SITB domain (non‐suicidal self‐harm vs. aggregate SITBs: *F*_1,11_ = 13.692, *p* = 0.004). Studies specifically focussing on non‐suicidal self‐harm reported *increased* electrodermal activity in participants who self‐harmed (Hedge's *g* = 0.249, SE = 0.085, 95% CI = [0.063; 0.436], *t* = 2.939, *p* = 0.014). In contrast, studies focussing on SITBs more broadly (both suicidal ideation and suicidal/non‐suicidal self‐harming behaviours), reported significantly *decreased* electrodermal activity in participants reporting SITBs compared to those who did not (Hedge's *g* = −0.567, SE = 0.153, 95% CI = [−0.905; −0.230], *t* = −3.700, *p* = 0.004). Note that we excluded one study from this analysis (Wang et al., 2021), since it was the only one focussing exclusively on suicide ideation/attempt.

The study without available data for the meta‐analysis (Crowell et al., 2012) found reduced skin conductance at baseline (but not during a task) in adolescents with SITBs versus controls.

## DISCUSSION

We carried out the first systematic review and meta‐analysis investigating the presence of autonomic dysregulation in children and young people displaying self‐injurious thoughts and behaviours (SITBs). Overall, we found that SITBs are associated with altered cardiac measures of arousal regulation, with a small‐to‐medium effect size. Results were particularly consistent for indices of heart rate variability (HRV), which were reduced in children and young people with SITBs, whereas we did not find significant effects for other cardiac measures. While findings for cardiac measures appeared consistent across different forms of SITBs, evidence for electrodermal measures appeared weaker and more preliminary, as an association with electrodermal measures did not emerge when considering all SITBs together and varied based on the investigated SITB domain. Specifically, children and young people with non‐suicidal self‐harm showed increased electrodermal activity, with a medium‐to‐large effect size, while reduced electrodermal activity was reported by studies not discriminating between different type of SITBs (i.e., combining groups of children and young people experiencing suicidal ideation, suicidal self‐harm and non‐suicidal self‐harm), with a small‐to‐medium effect size. As most studies did not distinguish between different forms of SITBs, future research is needed to clarify the association between SITBs and electrodermal indices of arousal.

There is significant interest in the possible application of objective measures of ANS regulation as biomarkers of clinical conditions, including SITBs and emotional dysregulation. To move towards this direction, further research should investigate the predictive utility of autonomic dysregulation for children and young people. Our meta‐analysis showed no effect of age, suggesting that altered autonomic functioning may characterise individuals displaying SITBs from childhood to young adulthood. Yet, as the majority the reviewed studies reported cross‐sectional group comparisons, primarily during adolescence, future longitudinal research should test whether autonomic dysregulation in those experiencing SITBs persists later in life and how accurately it predicts death by suicide. Such future studies could lead to the development of new tools, based on both self‐ and parent‐report and autonomic measures, showing sufficient specificity and sensitivity for detecting people at high suicide risk. Furthermore, it will be important to identify protective factors promoting improvements in autonomic function and reduction of SITBs, with an eye to suicide prevention, and to understand whether pharmacological (e.g., antidepressants) and/or non‐pharmacological interventions (e.g., dialectic behavioural therapy) lead – concurrently – to changes in autonomic functioning and reduction of SITBs severity.

Our findings partly support previous literature suggesting that SITBs may have a self‐regulatory function (Glenn et al., 2018; van Hoorn, 2020). A possible interpretation is that atypical arousal regulation (e.g., due to altered functioning of central and peripheral autonomic systems) is associated with the onset of SITBs, which could be implemented as maladaptive self‐regulation strategies. Previous studies, in fact, found a link between SITBs (particularly non‐suicidal self‐harm) and emotion dysregulation (Bridge et al., 2006; Edmondson et al., 2016), suggesting that self‐harm may represent an attempt to regulate emotion and affect, or to “feel something” other than numbness, resulting in calmness, relaxation and improved emotion regulation (van Hoorn, 2020). More research is needed to understand the causal relationship (if any) between self‐harm and autonomic dysfunction, and whether engaging in SITBs produces subsequent effects on the ANS (even temporarily).

Just over half of the studies included participants with a range of psychiatric diagnoses, in addition to SITBs. Considering that SITBs and associated autonomic dysregulation may also characterise individuals without a clinical diagnosis of psychiatric conditions (Fox et al., 2018; O’Connor, 2021), this underscores the importance of investigating SITBs in transdiagnostic samples, instead of focussing on populations with a specific condition or formal diagnosis. Arousal dysregulation has also been reported in children and young people with psychiatric conditions commonly associated with SITBs, such as mood and neurodevelopmental disorders (Bellato et al., 2020; Michelini et al., 2021), and it has been found associated with emotion dysregulation (Bellato et al., 2023). Future studies should elucidate whether ANS dysfunction represents a shared mechanism underlying these different conditions, or whether associations with psychiatric conditions may be explained by co‐occurring SITBs (or vice‐versa).

The present study has some limitations. Especially in relation to electrodermal measures, we could only include a limited number of studies in the meta‐analysis, even though our search was broad enough to capture the existing literature, limiting the statistical power of our analyses. Several studies were rated as being of poor quality (about 55%) and had methodological flaws, especially in relation to the methods used to ascertain the presence of SITBs (often based on self‐ or parent‐report). However, cross‐study heterogeneity was significant only for the meta‐analysis on cardiac measures, but not when considering the effect of type of cardiac measure (*Q* = 36.9927, *p* = 0.0952), and it was not significant for the meta‐analysis on electrodermal measures. Moreover, publication bias was detected for the meta‐analysis on electrodermal measures but the trim and fill analysis we conducted estimated that no study was missing due to this type of bias; this was probably due to the reduced number of studies existing in the literature, included in our meta‐analysis, highlighting that more empirical studies are needed to clarify the precise associations between electrodermal activity and different SITB domains.

## CONCLUSION

Our systematic review and meta‐analysis found evidence of reduced parasympathetic regulation as well as more tentative evidence of altered electrodermal activity in children and young people displaying self‐injurious thoughts and behaviours. Future longitudinal studies in heterogeneous samples are needed to further clarify the developmental relationship between autonomic dysregulation and the onset of SITBs, as well as future suicide. This will be helpful for developing tools capable of identifying children and young people at highest risk of engaging in self‐harming behaviours and ultimately reduce the number of individuals dying by suicide.

## AUTHOR CONTRIBUTIONS

**Alessio Bellato**: Conceptualization, Data curation, Formal analysis, Investigation, Methodology, Project administration, Resources, Software, Validation, Visualization, Writing – original draft, Writing – review & editing. **Muskaan Aleeza Admani**: Data curation, Writing – review & editing. **Camila Deak**: Data curation, Writing – review & editing. **Luis Carlos Farhat**: Data curation, Writing – review & editing. **Maria Carolina Fontana Antunes de Oliveira**: Data curation, Writing – review & editing. **Rebeca Vasconcelos**: Data curation, Writing – review & editing. **Margherita Malanchini**: Writing – review & editing. **Elizabeth Shepard**: Conceptualization, Investigation, Methodology, Project administration, Supervision, Writing – original draft, Writing – review & editing. **Giorgia Michelini**: Conceptualization, Investigation, Methodology, Project administration, Supervision, Writing – original draft, Writing – review & editing.

## CONFLICTS OF INTEREST STATEMENT

Elizabeth Shephard and Giorgia Michelini both serve on the JCPP Advances Editorial Advisory Board. The remaining authors declare they have no competing or potential conflicts of interest.

## ETHICAL CONSIDERATIONS

Based on the nature of the present study (systematic review and meta‐analysis of already published literature), ethical approval was not sought from the authors' institution.

## Supporting information

Supporting Information S1

## Data Availability

Raw data inputted in the meta‐analyses are available at the following link: https://osf.io/qdght/.
