# Supplementary material for: Autonomic dysregulation and self‐injurious thoughts and behaviours in children and young people: A systematic review and meta‐analysis
Source: JCPP Adv. 2023 Mar 23;3(3):e12148. doi: 10.1002/jcv2.12148 (PMC10501700; doi:10.1002/jcv2.12148)

**Supporting Information**

**Bellato et al., “Autonomic dysregulation and self-injurious thoughts and behaviours (SITBs) in children and young people: a systematic review and meta-analysis”**

[Appendix S1: PRISMA Checklist 2](#_Toc112837680)

[Appendix S2: Search strategy (last search: 10^th^ May 2022); Search details, inclusion criteria and data extraction details 7](#_Toc112837681)

[Appendix S3. Changes to the original protocol, with reasons for the changes 8](#_Toc112837682)

[Appendix S4. List of excluded papers after full-text screening 9](#_Toc112837683)

[Appendix S5. Quality appraisal of studies included in the review 20](#_Toc112837684)

[Table S1. Newcastle-Ottawa Scale for Cross-Sectional Studies 20](#_Toc112837685)

[Table S2. Newcastle-Ottawa Scale for Cohort Studies 22](#_Toc112837686)

[Appendix S6. Meta-analysis on cardiac measures 23](#_Toc112837687)

[Table S3. Summary of studies investigating the association between cardiac measures and SITBs 23](#_Toc112837688)

[Figure S1. Funnel plot assessing publication bias for studies investigating the association between cardiac measures and SITBs. 25](#_Toc112837689)

[Appendix S7. Meta-analysis on electrodermal measures 26](#_Toc112837690)

[Table S4. Summary of studies investigating the association between electrodermal measures and SITBs 26](#_Toc112837691)

[Figure S2. Funnel plot assessing publication bias for studies investigating the association between electrodermal activity and SITBs. 28](#_Toc112837692)

# Appendix S1: PRISMA Checklist

| **Section and Topic** | **Item #** | **Checklist item** | **Location where item is reported** |
| --- | --- | --- | --- |
| **TITLE** | | |  |
| Title | 1 | Identify the report as a systematic review. | Title |
| **ABSTRACT** | | |  |
| Abstract | 2 | See the PRISMA 2020 for Abstracts checklist. | Abstract |
| **INTRODUCTION** | | |  |
| Rationale | 3 | Describe the rationale for the review in the context of existing knowledge. | Introduction |
| Objectives | 4 | Provide an explicit statement of the objective(s) or question(s) the review addresses. | Introduction |
| **METHODS** | | |  |
| Eligibility criteria | 5 | Specify the inclusion and exclusion criteria for the review and how studies were grouped for the syntheses. | Methods, paragraph “Search strategy and selection criteria” |
| Information sources | 6 | Specify all databases, registers, websites, organisations, reference lists and other sources searched or consulted to identify studies. Specify the date when each source was last searched or consulted. | Methods, paragraph “Search strategy and selection criteria” |
| Search strategy | 7 | Present the full search strategies for all databases, registers and websites, including any filters and limits used. | Supplement 2 |
| Selection process | 8 | Specify the methods used to decide whether a study met the inclusion criteria of the review, including how many reviewers screened each record and each report retrieved, whether they worked independently, and if applicable, details of automation tools used in the process. | Methods, paragraph “Data selection, extraction and coding” |
| Data collection process | 9 | Specify the methods used to collect data from reports, including how many reviewers collected data from each report, whether they worked independently, any processes for obtaining or confirming data from study investigators, and if applicable, details of automation tools used in the process. | Methods, paragraph “Data selection, extraction and coding” |
| Data items | 10a | List and define all outcomes for which data were sought. Specify whether all results that were compatible with each outcome domain in each study were sought (e.g. for all measures, time points, analyses), and if not, the methods used to decide which results to collect. | Methods, paragraphs “Data selection, extraction and coding” and “Outcomes and assessment of study quality” |
|  | 10b | List and define all other variables for which data were sought (e.g. participant and intervention characteristics, funding sources). Describe any assumptions made about any missing or unclear information. | Methods, paragraph “Data selection, extraction and coding” |
| Study risk of bias assessment | 11 | Specify the methods used to assess risk of bias in the included studies, including details of the tool(s) used, how many reviewers assessed each study and whether they worked independently, and if applicable, details of automation tools used in the process. | Methods, paragraph “Outcomes and assessment of study quality” and Supplement 5 |
| Effect measures | 12 | Specify for each outcome the effect measure(s) (e.g. risk ratio, mean difference) used in the synthesis or presentation of results. | Methods, paragraph “Data synthesis and analysis” |
| Synthesis methods | 13a | Describe the processes used to decide which studies were eligible for each synthesis (e.g. tabulating the study intervention characteristics and comparing against the planned groups for each synthesis (item #5)). | Methods, paragraph “Data synthesis and analysis” |
|  | 13b | Describe any methods required to prepare the data for presentation or synthesis, such as handling of missing summary statistics, or data conversions. | Methods, paragraph “Data synthesis and analysis” |
|  | 13c | Describe any methods used to tabulate or visually display results of individual studies and syntheses. | Methods, paragraph “Data synthesis and analysis” |
|  | 13d | Describe any methods used to synthesize results and provide a rationale for the choice(s). If meta-analysis was performed, describe the model(s), method(s) to identify the presence and extent of statistical heterogeneity, and software package(s) used. | Methods, paragraph “Data synthesis and analysis” |
|  | 13e | Describe any methods used to explore possible causes of heterogeneity among study results (e.g. subgroup analysis, meta-regression). | Methods, paragraph “Data synthesis and analysis” |
|  | 13f | Describe any sensitivity analyses conducted to assess robustness of the synthesized results. | n/a. No sensitivity analyses conducted. |
| Reporting bias assessment | 14 | Describe any methods used to assess risk of bias due to missing results in a synthesis (arising from reporting biases). | Methods, paragraph “Outcomes and assessment of study quality” and Supplement 5 |
| Certainty assessment | 15 | Describe any methods used to assess certainty (or confidence) in the body of evidence for an outcome. | n/a |
| **RESULTS** | | |  |
| Study selection | 16a | Describe the results of the search and selection process, from the number of records identified in the search to the number of studies included in the review, ideally using a flow diagram. | Results and Figure 1 |
|  | 16b | Cite studies that might appear to meet the inclusion criteria, but which were excluded, and explain why they were excluded. | Supplement 4 |
| Study characteristics | 17 | Cite each included study and present its characteristics. | Table 1 |
| Risk of bias in studies | 18 | Present assessments of risk of bias for each included study. | Supplement 5 |
| Results of individual studies | 19 | For all outcomes, present, for each study: (a) summary statistics for each group (where appropriate) and (b) an effect estimate and its precision (e.g. confidence/credible interval), ideally using structured tables or plots. | Tables 2 and 3; Figures 2 and 4 |
| Results of syntheses | 20a | For each synthesis, briefly summarise the characteristics and risk of bias among contributing studies. | Supplement 5 |
|  | 20b | Present results of all statistical syntheses conducted. If meta-analysis was done, present for each the summary estimate and its precision (e.g. confidence/credible interval) and measures of statistical heterogeneity. If comparing groups, describe the direction of the effect. | Results, Figure 2 and 4 |
|  | 20c | Present results of all investigations of possible causes of heterogeneity among study results. | Results, Figure 3 and 5 |
|  | 20d | Present results of all sensitivity analyses conducted to assess the robustness of the synthesized results. | Results |
| Reporting biases | 21 | Present assessments of risk of bias due to missing results (arising from reporting biases) for each synthesis assessed. | Supplement 5 |
| Certainty of evidence | 22 | Present assessments of certainty (or confidence) in the body of evidence for each outcome assessed. | n/a |
| **DISCUSSION** | | |  |
| Discussion | 23a | Provide a general interpretation of the results in the context of other evidence. | Discussion |
|  | 23b | Discuss any limitations of the evidence included in the review. | Discussion |
|  | 23c | Discuss any limitations of the review processes used. | Discussion |
|  | 23d | Discuss implications of the results for practice, policy, and future research. | Discussion |
| **OTHER INFORMATION** | | |  |
| Registration and protocol | 24a | Provide registration information for the review, including register name and registration number, or state that the review was not registered. | Abstract, Methods |
|  | 24b | Indicate where the review protocol can be accessed, or state that a protocol was not prepared. | Abstract, Methods |
|  | 24c | Describe and explain any amendments to information provided at registration or in the protocol. | Supplement 3 |
| Support | 25 | Describe sources of financial or non-financial support for the review, and the role of the funders or sponsors in the review. | Acknowledgments |
| Competing interests | 26 | Declare any competing interests of review authors. | Acknowledgments |
| Availability of data, code and other materials | 27 | Report which of the following are publicly available and where they can be found: template data collection forms; data extracted from included studies; data used for all analyses; analytic code; any other materials used in the review. | Methods, paragraph “Data synthesis and analysis” |

*From:*  Page MJ, McKenzie JE, Bossuyt PM, Boutron I, Hoffmann TC, Mulrow CD, et al. The PRISMA 2020 statement: an updated guideline for reporting systematic reviews. BMJ 2021;372:n71. doi: 10.1136/bmj.n71

# Appendix S2: Search strategy (last search: 10^th^ May 2022); Search details, inclusion criteria and data extraction details

(suicid* OR self-harm OR self-injury OR self-cut OR self-inflict OR self-mutilate OR self-damage) AND (((autonomic arousal) OR (arousal) OR (autonomic nervous system) OR (ANS) OR (physiological arousal) OR (pupil* reflex) OR (pupil size) OR (pupil diameter) OR (pupillometry) OR (heart rate) OR (heart rate variability) OR (preejection period) OR (pre-ejection period) OR (respiratory sinus arrhythmia) OR (vagal tone) OR (galvanic skin response) OR (electrodermal activity) OR (electro-dermal activity) OR (electrodermal response) OR (electro-dermal response) OR (skin conductance) OR (psychogalvanic response) OR (sympathetic skin response)))

Keywords for these domains were selected based on whether they were used in previous systematic review and/or meta-analyses. Corresponding authors of conference abstracts deemed potentially eligible were contacted to request additional information on study eligibility and, if needed, data for the meta-analysis. No language or time restrictions were applied.

We included cross-sectional and cohort studies that:

1. reported indices of autonomic arousal;
2. included a sample comprised of individuals aged 0-25 years with SITBs
3. SITBs were ascertained either via psychometric instruments of self-harm/self-injury or clinical assessment)

In relation to cohort studies (without a control group), they should have:

1. investigated associations between scores on standardised scales assessing self-harm/injury and measures of autonomic functioning.

All studies we included in our systematic review were also included in previously published systematic reviews (Kang et al., 2020; Sarchiapone et al., 2018), in addition to more recent ones.

All studies included in previous systematic reviews (Kang et al., 2020; Sarchiapone et al., 2018) were included in this study, along with additional ones.

Extracted information included: study design, sample characteristics (sample size, age, sex distribution, racial/ethnic background), outcome measures (self-harm/suicidality scale utilized, if applicable; ANS measure analysed and method, main results and effect sizes with confidence intervals, when included in the article). Data not available from the manuscript were requested from study corresponding, first or senior authors.

Microsoft Excel and Endnote were used for screening and identification of duplicates.

# Appendix S3. Changes to the original protocol, with reasons for the changes

The original protocol was submitted on PROSPERO on 26^th^ April 2022: <https://www.crd.york.ac.uk/PROSPERO/display_record.php?RecordID=327605>.

On 31^st^ May 2022 we decided to not include cortisol or other biochemical measures of HPA functioning, since they are considered, in the literature, only indirect indices of autonomic arousal, and considering that previous systematic reviews and meta-analyses have been already published on this type of outcome measures (see, for example, Hernández-Díaz et al., 2020, <https://doi.org/10.1016/j.psychres.2020.113448>; and O'Connor et al., 2016, <https://doi.org/10.1016/j.psyneuen.2015.10.011>).

# Appendix S4. List of excluded papers after full-text screening

| **Reference** | **Main reason for exclusion** |
| --- | --- |
| Adolph, D., Teismann, T., Forkmann, T., Wannemüller, A., & Margraf, J. (2018). High frequency heart rate variability: Evidence for a transdiagnostic association with suicide ideation. Biol Psychol, 138, 165-171. https://doi.org/10.1016/j.biopsycho.2018.09.006 | Not focused on children, adolescents or young adults |
| Alharbi, R., Varese, F., Husain, N., & Taylor, P. J. (2020). Posttraumatic stress symptomology and non-suicidal self-injury: The role of intrusion and arousal symptoms. J Affect Disord, 276, 920-926. https://doi.org/10.1016/j.jad.2020.07.084 | Did not measure autonomic arousal |
| Amiri, A. M., Abtahi, M., Rabasco, A., Armey, M., Mankodiya, K., & Ieee. (2016). Emotional Reactivity Monitoring Using Electrodermal Activity Analysis In Individuals With Suicidal Behaviors 2016 10TH INTERNATIONAL SYMPOSIUM ON MEDICAL INFORMATION AND COMMUNICATION TECHNOLOGY (ISMICT), | Not focused on children, adolescents or young adults |
| Ashton, C. H., Marshall, E. F., Hassanyeh, F., Marsh, V. R., & Wright-Honari, S. (1994). Biological correlates of deliberate self-harm behaviour: a study of electroencephalographic, biochemical and psychological variables in parasuicide. Acta Psychiatr Scand, 90(5), 316-323. https://doi.org/10.1111/j.1600-0447.1994.tb01600.x | Not focused on children, adolescents or young adults |
| Bendezú, J. J., Calhoun, C. D., Patterson, M. W., Findley, A., Rudolph, K. D., Hastings, P., Nock, M. K., & Prinstein, M. J. (2021). Adolescent girls' stress responses as prospective predictors of self-injurious thoughts and behaviors: A person-centered, multilevel study. Dev Psychopathol, 1-21. https://doi.org/10.1017/s0954579420002229 | Did not measure autonomic arousal |
| Brain, K. L., Haines, J., & Williams, C. L. (1998). The psychophysiology of self-mutilation: Evidence of tension reduction. ARCHIVES OF SUICIDE RESEARCH, 4(3), 227-242. https://doi.org/10.1080/13811119808258298 | Not focused on children, adolescents or young adults |
| Carli, V., Hadlaczky, G., Petros, N. G., Iosue, M., Zeppegno, P., Gramaglia, C., Amore, M., Baca-Garcia, E., Batra, A., Cosman, D., Courtet, P., Di Sciascio, G., Ekstrand, J., Galfalvy, H., Gusmão, R., Jesus, C., Heitor, M. J., Constante, M., Rad, P. M., . . . Sarchiapone, M. (2021). A Naturalistic, European Multi-Center Clinical Study of Electrodermal Reactivity and Suicide Risk Among Patients With Depression. Front Psychiatry, 12, 765128. https://doi.org/10.3389/fpsyt.2021.765128 | Not focused on children, adolescents or young adults |
| Chang, C. C., Tzeng, N. S., Kao, Y. C., Yeh, C. B., & Chang, H. A. (2017). The relationships of current suicidal ideation with inflammatory markers and heart rate variability in unmedicated patients with major depressive disorder. Psychiatry Res, 258, 449-456. https://doi.org/10.1016/j.psychres.2017.08.076 | Not focused on children, adolescents or young adults |
| Chang, H. A., Chang, C. C., Chen, C. L., Kuo, T. B. J., Lu, R. B., & Huang, S. Y. (2012). Major depression is associated with cardiac autonomic dysregulation. ACTA NEUROPSYCHIATRICA, 24(6), 318-327. https://doi.org/10.1111/j.1601-5215.2011.00647.x | Not focused on children, adolescents or young adults |
| Chang, H. A., Chang, C. C., Chen, C. L., Kuo, T. B., Lu, R. B., & Huang, S. Y. (2013). Heart rate variability in patients with fully remitted major depressive disorder. Acta Neuropsychiatr, 25(1), 33-42. https://doi.org/10.1111/j.1601-5215.2012.00658.x | Not focused on children, adolescents or young adults |
| Chang, S. S., Bjørngaard, J. H., Tsai, M. K., Bjerkeset, O., Wen, C. P., Yip, P. S., Tsao, C. K., & Gunnell, D. (2016). Heart rate and suicide: findings from two cohorts of 533 000 Taiwanese and 75 000 Norwegian adults. Acta Psychiatr Scand, 133(4), 277-288. https://doi.org/10.1111/acps.12513 | Not focused on children, adolescents or young adults |
| Crowell, S. E., Baucom, B. R., McCauley, E., Potapova, N. V., Fitelson, M., Barth, H., Smith, C. J., & Beauchaine, T. P. (2013). Mechanisms of contextual risk for adolescent self-injury: invalidation and conflict escalation in mother-child interactions. *J Clin Child Adolesc Psychol*, *42*(4), 467-480. https://doi.org/10.1080/15374416.2013.785360 | Not focused on self-harm or suicide |
| Crowell, S. E., Baucom, B. R., Yaptangco, M., Bride, D., Hsiao, R., McCauley, E., & Beauchaine, T. P. (2014). Emotion dysregulation and dyadic conflict in depressed and typical adolescents: evaluating concordance across psychophysiological and observational measures. Biol Psychol, 98, 50-58. https://doi.org/10.1016/j.biopsycho.2014.02.009 | Not focused on self-harm or suicide |
| Crowell, S. E., Butner, J. E., Wiltshire, T. J., Munion, A. K., Yaptangco, M., & Beauchaine, T. P. (2017). Evaluating Emotional and Biological Sensitivity to Maternal Behavior Among Self-Injuring and Depressed Adolescent Girls Using Nonlinear Dynamics. *CLINICAL PSYCHOLOGICAL SCIENCE*, *5*(2), 272-285. https://doi.org/10.1177/2167702617692861 | Not focused on self-harm or suicide |
| Daurio, A., Ennis, C., & Taylor, J. (2019). SEXUAL MINORITY STATUS AND MOOD INDUCED PHYSIOLOGICAL RESPONSE IN FEMALES WHO ENGAGE IN NONSUICIDAL SELF-INJURY. Psychophysiology, 56, S51-S51. | No neurotypical controls, or the association between self-harm/suicidality and autonomic functioning was not investigated |
| Di Pierro, R., Sarno, I., Gallucci, M., & Madeddu, F. (2014). Nonsuicidal self-injury as an affect-regulation strategy and the moderating role of impulsivity. Child Adolesc Ment Health, 19(4), 259-264. https://doi.org/10.1111/camh.12063 | Did not measure autonomic arousal |
| Doron, A., Stein, D., Levine, Y., Abramovitch, Y., Eilat, E., & Neuman, M. (1998). Physiological reactions to a suicide film: suicide attempters, suicide ideators, and nonsuicidal patients. Suicide Life Threat Behav, 28(3), 309-314. https://onlinelibrary.wiley.com/doi/abs/10.1111/j.1943-278X.1998.tb00860.x?sid=nlm%3Apubmed | Not focused on children, adolescents or young adults |
| Duprey, E. B., Oshri, A., Liu, S., Kogan, S. M., & Caughy, M. O. (2021). Physiological Stress Response Reactivity Mediates the Link Between Emotional Abuse and Youth Internalizing Problems. Child Psychiatry Hum Dev, 52(3), 450-463. https://doi.org/10.1007/s10578-020-01033-1 | Not focused on self-harm or suicide |
| Edman, G., Asberg, M., Levander, S., & Schalling, D. (1986). Skin conductance habituation and cerebrospinal fluid 5-hydroxyindoleacetic acid in suicidal patients. Arch Gen Psychiatry, 43(6), 586-592. https://doi.org/10.1001/archpsyc.1986.01800060080010 | Not focused on children, adolescents or young adults |
| Fitzpatrick, S., Zeifman, R., Krantz, L., McMain, S., & Kuo, J. R. (2020). Getting Specific about Emotion and Self-Inflicted Injury: An Examination Across Emotion Processes in Borderline Personality Disorder. Arch Suicide Res, 24(sup1), 102-123. https://doi.org/10.1080/13811118.2019.1586605 | Not focused on children, adolescents or young adults |
| Forkmann, T., Meessen, J., Teismann, T., Sütterlin, S., Gauggel, S., & Mainz, V. (2016). Resting vagal tone is negatively associated with suicide ideation. J Affect Disord, 194, 30-32. https://doi.org/10.1016/j.jad.2016.01.032 | Not focused on children, adolescents or young adults |
| Fountoulakis, K. N., Iacovides, A., Fotiou, F., Nimatoudis, J., Bascialla, F., Ioannidou, C., Kaprinis, G., & Bech, P. (2004). Neurobiological and psychological correlates of suicidal attempts and thoughts of death in patients with major depression. Neuropsychobiology, 49(1), 42-52. https://doi.org/10.1159/000075338 | Not focused on children, adolescents or young adults |
| Franklin, J. C., Hessel, E. T., Aaron, R. V., Arthur, M. S., Heilbron, N., & Prinstein, M. J. (2010). The functions of nonsuicidal self-injury: support for cognitive-affective regulation and opponent processes from a novel psychophysiological paradigm. J Abnorm Psychol, 119(4), 850-862. https://doi.org/10.1037/a0020896 | Did not measure autonomic arousal |
| Freeman, R. L., Horner, R. H., & Reichle, J. (1999). Relation between heart rate and problem behaviors. Am J Ment Retard, 104(4), 330-345. https://doi.org/10.1352/0895-8017(1999)104<0330:Rbhrap>2.0.Co;2 | Not an empirical study |
| Funkhouser, C. J., Correa, K. A., Carrillo, V. L., Klemballa, D. M., & Shankman, S. A. (2019). The time course of responding to aversiveness in females with a history of non-suicidal self-injury. Int J Psychophysiol, 141, 1-8. https://doi.org/10.1016/j.ijpsycho.2019.04.008 | Not focused on children, adolescents or young adults |
| Garland, E. L., Riquino, M. R., Priddy, S. E., & Bryan, C. J. (2017). Suicidal ideation is associated with individual differences in prescription opioid craving and cue-reactivity among chronic pain patients. J Addict Dis, 36(1), 23-29. https://doi.org/10.1080/10550887.2016.1220800 | Not focused on children, adolescents or young adults |
| Giner-Bartolome, C., Mallorquí-Bagué, N., Tolosa-Sola, I., Steward, T., Jimenez-Murcia, S., Granero, R., & Fernandez-Aranda, F. (2017). Non-suicidal Self-Injury in Eating Disordered Patients: Associations with Heart Rate Variability and State-Trait Anxiety. Front Psychol, 8, 1163. https://doi.org/10.3389/fpsyg.2017.01163 | Not focused on children, adolescents or young adults |
| Glenn, C. R., Blumenthal, T. D., Klonsky, E. D., & Hajcak, G. (2011). Emotional reactivity in nonsuicidal self-injury: divergence between self-report and startle measures. Int J Psychophysiol, 80(2), 166-170. https://doi.org/10.1016/j.ijpsycho.2011.02.016 | Did not measure autonomic arousal |
| Grant, J. E., Redden, S. A., & Chamberlain, S. R. (2017). Cold pressor pain in skin picking disorder. Psychiatry Res, 249, 35-38. https://doi.org/10.1016/j.psychres.2016.12.050 | Not focused on children, adolescents or young adults |
| Gratz, K. L., & Roemer, L. (2008). The relationship between emotion dysregulation and deliberate self-harm among female undergraduate students at an urban commuter university. Cogn Behav Ther, 37(1), 14-25. https://doi.org/10.1080/16506070701819524 | Not focused on children, adolescents or young adults |
| Gratz, K. L., Richmond, J. R., Dixon-Gordon, K. L., Chapman, A. L., & Tull, M. T. (2019). Multimodal Assessment of Emotional Reactivity and Regulation in Response to Social Rejection Among Self-Harming Adults With and Without Borderline Personality Disorder. PERSONALITY DISORDERS-THEORY RESEARCH AND TREATMENT, 10(5), 395-405. https://doi.org/10.1037/per0000334 | Not focused on children, adolescents or young adults |
| Grove, J. L., Carlson, S. E., Parkhurst, K. A., & Smith, T. W. (2021). The role of pre-sleep arousal in the connection between insomnia and suicide risk. Death Stud, 1-7. https://doi.org/10.1080/07481187.2021.1964109 | Not focused on children, adolescents or young adults |
| Gupta, M., & Jarosz, P. (2017). Rapid Eye Movement (REM) Sleep is Associated with Increased Parasympathetic Tone and Decreased Suicidal Behavior in Posttraumatic Stress Disorder (PTSD). BIOLOGICAL PSYCHIATRY, 81(10), S337-S337. https://doi.org/10.1016/j.biopsych.2017.02.898 | Not focused on children, adolescents or young adults |
| Haines, J., Williams, C. L., Brain, K. L., & Wilson, G. V. (1995). The psychophysiology of self-mutilation. J Abnorm Psychol, 104(3), 471-489. https://doi.org/10.1037//0021-843x.104.3.471 | Not possible to determine final eligibility |
| Huang 2013 | Not focused on self-harm or suicide |
| Jandl, M., Steyer, J., & Kaschka, W. P. (2007). Suicide risk markers in major depression disorder (MDD): A study on electrodermal activity (EDA) and event related potentials (ERP). BIOLOGICAL PSYCHIATRY, 61(8), 204S-204S. | Not an empirical study |
| Jandl, M., Steyer, J., & Kaschka, W. P. (2010). Suicide risk markers in major depressive disorder: a study of electrodermal activity and event-related potentials. J Affect Disord, 123(1-3), 138-149. https://doi.org/10.1016/j.jad.2009.09.011 | Not focused on children, adolescents or young adults |
| Jokinen, J., Ouda, J., & Nordström, P. (2010). Noradrenergic function and HPA axis dysregulation in suicidal behaviour. Psychoneuroendocrinology, 35(10), 1536-1542. https://doi.org/10.1016/j.psyneuen.2010.05.008 | Did not measure autonomic arousal |
| Jones, I. H. (1982). Self-injury: toward a biological basis. Perspect Biol Med, 26(1), 137-150. https://doi.org/10.1353/pbm.1982.0006 | Not an empirical study |
| Kaschka, W., Jandl, M., & Steyer, J. (2006). Electrodermal activity (EDA) and event related potentials (ERP) as markers for suicide risk in depression. INTERNATIONAL JOURNAL OF NEUROPSYCHOPHARMACOLOGY, 9, S111-S112. | Not focused on children, adolescents or young adults |
| Kaufman, E. A., Crowell, S. E., Coleman, J., Puzia, M. E., Gray, D. D., & Strayer, D. L. (2018). Electroencephalographic and cardiovascular markers of vulnerability within families of suicidal adolescents: A pilot study. Biol Psychol, 136, 46-56. https://doi.org/10.1016/j.biopsycho.2018.05.007 | Not possible to determine final eligibility |
| Keller, F., Wolfersdorf, M., Straub, R., & Hole, G. (1991). Suicidal behaviour and electrodermal activity in depressive inpatients. Acta Psychiatr Scand, 83(5), 324-328. https://doi.org/10.1111/j.1600-0447.1991.tb05549.x | Not focused on children, adolescents or young adults |
| Khandoker, A. H., Luthra, V., Abouallaban, Y., Saha, S., Ahmed, K. I., Mostafa, R., Chowdhury, N., & Jelinek, H. F. (2017). Predicting depressed patients with suicidal ideation from ECG recordings. Med Biol Eng Comput, 55(5), 793-805. https://doi.org/10.1007/s11517-016-1557-y | Not focused on children, adolescents or young adults |
| Kleiman, E. M., Bentley, K. H., Maimone, J. S., Lee, H. S., Kilbury, E. N., Fortgang, R. G., Zuromski, K. L., Huffman, J. C., & Nock, M. K. (2021). Can passive measurement of physiological distress help better predict suicidal thinking? Transl Psychiatry, 11(1), 611. https://doi.org/10.1038/s41398-021-01730-y | Not focused on children, adolescents or young adults |
| Kleiman, E., Millner, A. J., Joyce, V. W., Nash, C. C., Buonopane, R. J., & Nock, M. K. (2019). Using Wearable Physiological Monitors With Suicidal Adolescent Inpatients: Feasibility and Acceptability Study. JMIR MHEALTH AND UHEALTH, 7(9). https://doi.org/10.2196/13725 | No neurotypical controls, or the association between self-harm/suicidality and autonomic functioning was not investigated |
| Klonsky, E. D. (2009). The functions of self-injury in young adults who cut themselves: clarifying the evidence for affect-regulation. Psychiatry Res, 166(2-3), 260-268. https://doi.org/10.1016/j.psychres.2008.02.008 | Did not measure autonomic arousal |
| Koenig, J., & Kaess, M. (2018). Autonomic Nervous System Function and Emotion Regulation in Adolescent Non-Suicidal Self-Injury. BIOLOGICAL PSYCHIATRY, 83(9), S371-S372. https://doi.org/10.1016/j.biopsych.2018.02.955 | No neurotypical controls, or the association between self-harm/suicidality and autonomic functioning was not investigated |
| Koenig, J., Weise, S., Rinnewitz, L., Hoppstadter, T., Niederbaumer, M., Parzer, P., Resch, F., & Kaess, M. (2017). Longitudinal Covariance of Resting State Heart Rate Variability and Borderline Personality Disorder Symptoms in Adolescents with Non-Suicidal Self-Injury. BIOLOGICAL PSYCHIATRY, 81(10), S271-S272. https://doi.org/10.1016/j.biopsych.2017.02.1080 | No neurotypical controls, or the association between self-harm/suicidality and autonomic functioning was not investigated |
| Koenig, J., Weise, S., Rinnewitz, L., Parzer, P., Resch, F., & Kaess, M. (2017). LONGITUDINAL DEVELOPMENT OF HEART RATE VARIABILITY AND BORDERLINE PERSONALITY DISORDER SYMPTOMS IN ADOLESCENTS WITH NON-SUICIDAL SELF-INJURY. Psychophysiology, 54, S100-S100. | Data already reported in another paper included in the review |
| Kranzler, A., Fehling, K. B., Lindqvist, J., Brillante, J., Yuan, F., Gao, X., Miller, A. L., & Selby, E. A. (2018). An Ecological Investigation of the Emotional Context Surrounding Nonsuicidal Self-Injurious Thoughts and Behaviors in Adolescents and Young Adults. Suicide Life Threat Behav, 48(2), 149-159. https://doi.org/10.1111/sltb.12373 | Did not measure autonomic arousal |
| Kwon, A., Lee, H. S., & Lee, S. H. (2021). The Mediation Effect of Hyperarousal Symptoms on the Relationship Between Childhood Physical Abuse and Suicidal Ideation of Patients With PTSD. Front Psychiatry, 12, 613735. https://doi.org/10.3389/fpsyt.2021.613735 | Did not measure autonomic arousal |
| Law, K. C., & Anestis, M. D. (2021). Testing Whether Suicide Capability Has a Dynamic Propensity: The Role of Affect and Arousal on Momentary Fluctuations in Suicide Capability. Front Psychol, 12, 590187. https://doi.org/10.3389/fpsyg.2021.590187 | Not focused on children, adolescents or young adults |
| Lee, D., Baek, J. H., Cho, Y. J., & Hong, K. S. (2021). Association of Resting Heart Rate and Heart Rate Variability With Proximal Suicidal Risk in Patients With Diverse Psychiatric Diagnoses. Front Psychiatry, 12, 652340. https://doi.org/10.3389/fpsyt.2021.652340 | Not focused on children, adolescents or young adults |
| Lemogne, C., Thomas, F., Consoli, S. M., Pannier, B., Jégo, B., & Danchin, N. (2011). Heart rate and completed suicide: evidence from the IPC cohort study. Psychosom Med, 73(9), 731-736. https://doi.org/10.1097/PSY.0b013e3182365dc7 | Not focused on children, adolescents or young adults |
| Lester, D. (1991). Depression, suicidal ideation and autonomic reactivity. Percept Mot Skills, 73(1), 294. https://doi.org/10.2466/pms.1991.73.1.294 | Did not measure autonomic arousal |
| Lin, B., Kaliush, P. R., Conradt, E., Terrell, S., Neff, D., Allen, A. K., Smid, M. C., Monk, C., & Crowell, S. E. (2019). Intergenerational transmission of emotion dysregulation: Part I. Psychopathology, self-injury, and parasympathetic responsivity among pregnant women. Dev Psychopathol, 31(3), 817-831. https://doi.org/10.1017/s0954579419000336 | Not focused on children, adolescents or young adults |
| Lin, Y., Lin, C., Sun, I. W., Hsu, C. C., Fang, C. K., Lo, M. T., Huang, H. C., & Liu, S. I. (2015). Resting respiratory sinus arrhythmia is related to longer hospitalization in mood-disordered repetitive suicide attempters. World J Biol Psychiatry, 16(5), 323-333. https://doi.org/10.3109/15622975.2015.1017603 | Not focused on children, adolescents or young adults |
| McCall, W. V., Sareddy, S., Youssef, N. A., Miller, B. J., & Rosenquist, P. B. (2021). The pupillary light reflex as a point-of-care test for suicide risk: Preliminary results. Psychiatry Res, 295, 113582. https://doi.org/10.1016/j.psychres.2020.113582 | Not focused on children, adolescents or young adults |
| McGirr, A., Diaconu, G., Berlim, M. T., Pruessner, J. C., Sablé, R., Cabot, S., & Turecki, G. (2010). Dysregulation of the sympathetic nervous system, hypothalamic-pituitary-adrenal axis and executive function in individuals at risk for suicide. J Psychiatry Neurosci, 35(6), 399-408. https://doi.org/10.1503/jpn.090121 | Not focused on children, adolescents or young adults |
| Meerwijk, E. L., & Weiss, S. J. (2016). Does suicidal desire moderate the association between frontal delta power and psychological pain? PeerJ, 4, e1538. https://doi.org/10.7717/peerj.1538 | Not focused on children, adolescents or young adults |
| Mendez, A. J., Lado, M. J., Vila, X. A., Rodriguez-Linares, L., Alonso, R. A., & Garcia-Caballero, A. (2013). Heart of Darkness Heart Rate Variability on Patients with Risk of Suicide PROCEEDINGS OF THE 2013 8TH IBERIAN CONFERENCE ON INFORMATION SYSTEMS AND TECHNOLOGIES (CISTI 2013), | Not focused on children, adolescents or young adults |
| Miller, B. J., Sareddy, S., Rosenquist, P. B., & McCall, W. V. (2021). Pupillary light reflex markers of suicide risk in a trans-diagnostic sample. Schizophr Res, 235, 1-2. https://doi.org/10.1016/j.schres.2021.06.027 | Not focused on children, adolescents or young adults |
| Morabito, D. M., Boffa, J. W., Bedford, C. E., Chen, J. P., & Schmidt, N. B. (2020). Hyperarousal symptoms and perceived burdensomeness interact to predict suicidal ideation among trauma-exposed individuals. J Psychiatr Res, 130, 218-223. https://doi.org/10.1016/j.jpsychires.2020.07.029 | Not focused on children, adolescents or young adults |
| Naoum, J., Reitz, S., Krause-Utz, A., Kleindienst, N., Willis, F., Kuniss, S., Baumgärtner, U., Mancke, F., Treede, R. D., & Schmahl, C. (2016). The role of seeing blood in non-suicidal self-injury in female patients with borderline personality disorder. Psychiatry Res, 246, 676-682. https://doi.org/10.1016/j.psychres.2016.10.066 | Not focused on children, adolescents or young adults |
| Neacsiu, A. D., Fang, C. M., Rodriguez, M., & Rosenthal, M. Z. (2018). Suicidal Behavior and Problems with Emotion Regulation. Suicide Life Threat Behav, 48(1), 52-74. https://doi.org/10.1111/sltb.12335 | Not focused on children, adolescents or young adults |
| Ortiz, A., Bradler, K., Moorti, P., MacLean, S., Husain, M. I., Sanches, M., Goldstein, B. I., Alda, M., & Mulsant, B. H. (2021). Reduced heart rate variability is associated with higher illness burden in bipolar disorder. J Psychosom Res, 145, 110478. https://doi.org/10.1016/j.jpsychores.2021.110478 | Not focused on children, adolescents or young adults |
| Ortiz, A., Bradler, K., Moorti, P., MacLean, S., Ishrat Husain, M., Sanches, M., Goldstein, B. I., Alda, M., & Mulsant, B. H. (2022). Increased sympathetic tone is associated with illness burden in bipolar disorder. J Affect Disord, 297, 471-476. https://doi.org/10.1016/j.jad.2021.10.089 | Not focused on children, adolescents or young adults |
| Rechlin, T., Weis, M., Spitzer, A., & Kaschka, W. P. (1994). Are affective disorders associated with alterations of heart rate variability? J Affect Disord, 32(4), 271-275. https://doi.org/10.1016/0165-0327(94)90091-4 | Not focused on children, adolescents or young adults |
| Reitz, S., Kluetsch, R., Niedtfeld, I., Knorz, T., Lis, S., Paret, C., Kirsch, P., Meyer-Lindenberg, A., Treede, R. D., Baumgärtner, U., Bohus, M., & Schmahl, C. (2015). Incision and stress regulation in borderline personality disorder: neurobiological mechanisms of self-injurious behaviour. Br J Psychiatry, 207(2), 165-172. https://doi.org/10.1192/bjp.bp.114.153379 | Not focused on children, adolescents or young adults |
| Reitz, S., Krause-Utz, A., Pogatzki-Zahn, E. M., Ebner-Priemer, U., Bohus, M., & Schmahl, C. (2012). Stress regulation and incision in borderline personality disorder--a pilot study modeling cutting behavior. J Pers Disord, 26(4), 605-615. https://doi.org/10.1521/pedi.2012.26.4.605 | Not focused on children, adolescents or young adults |
| Ribeiro, J. D., Yen, S., Joiner, T., & Siegler, I. C. (2015). Capability for suicide interacts with states of heightened arousal to predict death by suicide beyond the effects of depression and hopelessness. J Affect Disord, 188, 53-59. https://doi.org/10.1016/j.jad.2015.07.037 | Did not measure autonomic arousal |
| Rinnewitz, L., Koenig, J., Parzer, P., Brunner, R., Resch, F., & Kaess, M. (2018). Childhood Adversity and Psychophysiological Reactivity to Pain in Adolescent Nonsuicidal Self-Injury. Psychopathology, 51(5), 346-352. https://doi.org/10.1159/000491702 | No neurotypical controls, or the association between self-harm/suicidality and autonomic functioning was not investigated |
| Rizk, M. M., Galfalvy, H., Singh, T., Keilp, J. G., Sublette, M. E., Oquendo, M. A., Mann, J. J., & Stanley, B. (2018). Toward subtyping of suicidality: Brief suicidal ideation is associated with greater stress response. JOURNAL OF AFFECTIVE DISORDERS, 230, 87-92. https://doi.org/10.1016/j.jad.2018.01.012 | Not focused on children, adolescents or young adults |
| Sikander, D., Arvaneh, M., Amico, F., Healy, G., Ward, T., Kearney, D., Mohedano, E., Fagan, J., Yek, J., Smeaton, A. F., Brophy, J., & Ieee. (2016). Predicting Risk of Suicide Using Resting State Heart Rate 2016 ASIA-PACIFIC SIGNAL AND INFORMATION PROCESSING ASSOCIATION ANNUAL SUMMIT AND CONFERENCE (APSIPA), | Not focused on children, adolescents or young adults |
| Song, B. A., Yoo, S. Y., Kang, H. Y., Byeon, S. H., Shin, S. H., Hwang, E. J., & Lee, S. H. (2011). Post-Traumatic Stress Disorder, Depression, and Heart-Rate Variability among North Korean Defectors. Psychiatry Investig, 8(4), 297-304. https://doi.org/10.4306/pi.2011.8.4.297 | Not focused on children, adolescents or young adults |
| Spiegel, D. (1969). Autonomic reactivity in relation to the affective meaning of suicide. J Clin Psychol, 25(4), 359-362. https://doi.org/10.1002/1097-4679(196910)25:4<359::aid-jclp2270250404>3.0.co;2-k | Not possible to determine final eligibility |
| Straub, R., Jandl, M., & Wolfersdorf, M. (2003). [Mental state and electrodermal activity in depressed patients during acute suicidal period]. Psychiatr Prax, 30 Suppl 2, S183-186. (Befindlichkeit und elektrodermale Aktivität depressiver Patienten während akuter Suizidalität.) | Not focused on children, adolescents or young adults |
| Suss, A., Homel, P., Wilson, T. E., & Shah, B. (2004). Risk factors for nonfatal suicide behaviors among inner-city adolescents. Pediatr Emerg Care, 20(7), 426-429. https://doi.org/10.1097/01.pec.0000134925.08868.42 | No neurotypical controls, or the association between self-harm/suicidality and autonomic functioning was not investigated |
| Thorell, L. H. (1987). Electrodermal activity in suicidal and nonsuicidal depressive patients and in matched healthy subjects. Acta Psychiatr Scand, 76(4), 420-430. https://doi.org/10.1111/j.1600-0447.1987.tb05627.x | Not focused on children, adolescents or young adults |
| Thorell, L. H., & D'Elia, G. (1987). Electrodermal responsivity and suicide risk. Arch Gen Psychiatry, 44(12), 1112. https://doi.org/10.1001/archpsyc.1987.01800240088017 | Not focused on children, adolescents or young adults |
| Thorell, L. H., & d'Elia, G. (1988). Electrodermal activity in depressive patients in remission and in matched healthy subjects. Acta Psychiatr Scand, 78(2), 247-253. https://doi.org/10.1111/j.1600-0447.1988.tb06332.x | Not focused on children, adolescents or young adults |
| Thorell, L. H., Kjellman, B. F., d'Elia, G., & Kågedal, B. (1988). Electrodermal activity in relation to cortisol dysregulation in depressive patients. Acta Psychiatr Scand, 78(6), 743-753. https://doi.org/10.1111/j.1600-0447.1988.tb06414.x | Not focused on children, adolescents or young adults |
| Tsypes, A., James, K. M., Woody, M. L., Feurer, C., Kudinova, A. Y., & Gibb, B. E. (2018). Resting respiratory sinus arrhythmia in suicide attempters. Psychophysiology, 55(2). https://doi.org/10.1111/psyp.12978 | Not focused on children, adolescents or young adults |
| Weinberg, A., & Klonsky, E. D. (2012). The effects of self-injury on acute negative arousal: A laboratory simulation. MOTIVATION AND EMOTION, 36(2), 242-254. https://doi.org/10.1007/s11031-011-9233-x | Did not measure autonomic arousal |
| Wiebenga, J. X. M., Heering, H. D., Eikelenboom, M., van Hemert, A. M., van Oppen, P., & Penninx, B. (2022). Associations of three major physiological stress systems with suicidal ideation and suicide attempts in patients with a depressive and/or anxiety disorder. Brain Behav Immun, 102, 195-205. https://doi.org/10.1016/j.bbi.2022.02.021 | Not focused on children, adolescents or young adults |
| Wilson, S. T., Chesin, M., Fertuck, E., Keilp, J., Brodsky, B., Mann, J. J., Sönmez, C. C., Benjamin-Phillips, C., & Stanley, B. (2016). Heart rate variability and suicidal behavior. Psychiatry Res, 240, 241-247. https://doi.org/10.1016/j.psychres.2016.04.033 | Not focused on children, adolescents or young adults |
| Wolfersdorf, M., & Straub, R. (1994). Electrodermal reactivity in male and female depressive patients who later died by suicide. Acta Psychiatr Scand, 89(4), 279-284. https://doi.org/10.1111/j.1600-0447.1994.tb01514.x | Not focused on children, adolescents or young adults |
| Wolfersdorf, M., Straub, R., & Barg, T. (1996). Electrodermal activity (EDA) and suicidal behavior. Crisis, 17(2), 69-77. https://doi.org/10.1027/0227-5910.17.2.69 | Not focused on children, adolescents or young adults |
| Wolfersdorf, M., Straub, R., & Hole, G. (1993). Electrodermal activity in depressive men and women with violent or non-violent suicide attempts. Schweiz Arch Neurol Psychiatr (1985), 144(2), 173-184. | Not focused on children, adolescents or young adults |
| Young, H. A., Davies, J., Freegard, G., & Benton, D. (2021). Nonsuicidal Self-Injury Is Associated With Attenuated Interoceptive Responses to Self-Critical Rumination. Behav Ther, 52(5), 1123-1136. https://doi.org/10.1016/j.beth.2021.02.010 | Not focused on children, adolescents or young adults |
| Zhu, X. Y., Gedeon, T., Caldwell, S., & Jones, R. (2019). Visceral versus Verbal: Can We See Depression? ACTA POLYTECHNICA HUNGARICA, 16(9), 113-133. https://doi.org/10.12700/APH.16.9.2019.9.7 | Not focused on children, adolescents or young adults |

# Appendix S5. Quality appraisal of studies included in the review

## Table S1. Newcastle-Ottawa Scale for Cross-Sectional Studies

|  | Chesin 2020 | Crowell 2005 | Crowell 2012 | James 2017 | Kaess 2012 | Kaufman 2020 | Koenig 2017 (J Psych Neurosc) |
| --- | --- | --- | --- | --- | --- | --- | --- |
| **Selection** |  |  |  |  |  |  |  |
| 1. Is the case definition adequate? | B | B | B | A* | B | A* | A* |
| 2. Representativeness of the cases | A* | B | B | A* | B | B | B |
| 3. Selection of controls | A* | A* | A* | A* | A* | A* | A* |
| 4. Definition of controls | B | A* | A* | A* | A* | A* | A* |
| **Comparability** |  |  |  |  |  |  |  |
| 5. Comparability of cases and controls on the basis of the design or analysis | * | * | * | ** | ** | * | ** |
| **Exposure** |  |  |  |  |  |  |  |
| 6. Ascertainment of exposure | D | C | D | D | D | C | A* |
| 7. Same method of ascertainment for cases and controls | A* | A* | A* | A* | A* | B | A* |
| 8. Non-response rate | A* | A* | A* | C | C | A* | A* |
| **Overall Quality** | **Fair** | **Fair** | **Fair** | **Poor** | **Poor** | **Poor** | **Good** |

|  | Koenig 2017 (Psych Res) | Nock 2008 | Tatnell 2018 | Tuna 2021 | Wang 2021 | Wielgus 2016 | Yang 2019 |
| --- | --- | --- | --- | --- | --- | --- | --- |
| **Selection** |  |  |  |  |  |  |  |
| 1. Is the case definition adequate? | A* | B | B | B | B | B | B |
| 2. Representativeness of the cases | B | B | B | B | B | B | B |
| 3. Selection of controls | C | A* | A* | A* | A* | A* | B |
| 4. Definition of controls | A* | A* | A* | A* | A* | A* | A* |
| **Comparability** |  |  |  |  |  |  |  |
| 5. Comparability of cases and controls on the basis of the design or analysis | ** | * | * | ** | * | * | None |
| **Exposure** |  |  |  |  |  |  |  |
| 6. Ascertainment of exposure | A* | B* | A* | C | C | B | A* |
| 7. Same method of ascertainment for cases and controls | A* | A* | A* | A* | A* | A* | A* |
| 8. Non-response rate | A* | C | C | C | C | A* | A* |
| **Overall Quality** | **Fair** | **Fair** | **Fair** | **Poor** | **Poor** | **Fair** | **Poor** |

## Table S2. Newcastle-Ottawa Scale for Cohort Studies

|  | Aldrich 2018 | Duprey 2019 | Fox 2018 | Giletta 2017 | Koenig 2018 | Sheridan 2021 |
| --- | --- | --- | --- | --- | --- | --- |
| **Selection** |  |  |  |  |  |  |
| 1. Representativeness of the exposed cohort | A* | A* | C | A* | C | B* |
| 2. Selection of the non-exposed cohort | A* | A* | B | N/A | N/A | A* |
| 3. Ascertainment of exposure | C | C | C | C | B* | A* |
| 4. Demonstration that outcome of interest was not present at start of study | A* | C | B | B | B | N/A |
| **Comparability** |  |  |  |  |  |  |
| 5. Comparability of cohorts on basis of design or analysis | ** | ** | None | None | None | None |
| **Outcome** |  |  |  |  |  |  |
| 6. Assessment of outcome | C | C | C | C | A* | A* |
| 7. Was follow-up long enough for outcomes to occur | A* | A* | N/A | N/A | A* | A* |
| 8. Adequacy of follow-up of cohort | A* | D | D | D | B* | D |
| **Overall Quality** | **Good** | **Poor** | **Poor** | **Poor** | **Poor** | **Poor** |

# Appendix S6. Meta-analysis on cardiac measures

## Table S3. Summary of studies investigating the association between cardiac measures and SITBs

| **Study** | **SITB domain** | **Developmental Stage** | **Cardiac measure** | **Experimental Task/Activity** | **Hedge's g** | **Variance (g)** | **Lower 95% CI** | **Upper 95% CI** |
| --- | --- | --- | --- | --- | --- | --- | --- | --- |
| Chesin, 2020 | Suicide ideation | Young adults | HRV reactivity | Arousal inducing tasks (Stroop Task and Cyberball) | -0.4897 | 0.0509 | -0.9319 | -0.0476 |
| Crowell, 2005.1 | All SITBs | Children/ Adolescents | PEP | Baseline/ Resting-state | -0.0923 | 0.0871 | -0.6706 | 0.486 |
| Crowell, 2005.2 | All SITBs | Children/ Adolescents | PEP | Sadness-evoking video | 0.0084 | 0.087 | -0.5696 | 0.5863 |
| Crowell, 2005.3 | All SITBs | Children/ Adolescents | RSA | Baseline/ Resting-state | -1.0878 | 0.1003 | -1.7085 | -0.4672 |
| Crowell, 2005.4 | All SITBs | Children/ Adolescents | RSA | Recovery to Sadness-evoking video | -1.2685 | 0.1051 | -1.9038 | -0.6332 |
| Duprey, 2019 | Suicide ideation | Young adults | HRV | Baseline/ Resting-state | -0.261 | 0.0248 | -0.5694 | 0.0473 |
| Fox, 2018.1 | Non-suicidal self-harm | Young adults | RSA | Baseline/ Resting-state | -0.1587 | 0.0573 | -0.628 | 0.3105 |
| Fox, 2018.2 | Non-suicidal self-harm | Young adults | RSA | Stress task (preparation of public speech and recovery) | -0.5546 | 0.0594 | -1.0323 | -0.077 |
| Giletta, 2017.1 | Suicide ideation | Children/ Adolescents | RSA | Baseline/ Resting-state | -0.2201 | 0.0305 | -0.5623 | 0.1222 |
| Giletta, 2017.2 | Suicide ideation | Children/ Adolescents | RSA | Modified Trier Social Stress Test | -0.7429 | 0.0324 | -1.0958 | -0.3901 |
| James, 2017.1 | Suicide ideation | Children/ Adolescents | RSA | Baseline/ Resting-state | -0.2086 | 0.0238 | -0.5111 | 0.0938 |
| James, 2017.2 | Suicide ideation | Children/ Adolescents | RSA | Vacation-planning task with parents | -0.3271 | 0.0239 | -0.6303 | -0.024 |
| James, 2017.3 | Suicide ideation | Children/ Adolescents | RSA | Discussion about issues with parents | -0.2522 | 0.0238 | -0.5548 | 0.0505 |
| Kaess, 2012 | Non-suicidal self-harm | Children/ Adolescents | HR reactivity | Trier Social Stress Test | -0.1914 | 0.1436 | -0.934 | 0.5512 |
| Kaufman, 2020.1 | All SITBs | Children/ Adolescents | RSA | Baseline/ Resting-state | -0.6877 | 0.0706 | 0.1669 | 1.2085 |
| Kaufman, 2020.2 | All SITBs | Children/ Adolescents | RSA | Social interaction with mother | -0.6877 | 0.0706 | 0.1669 | 1.2085 |
| Kaufman, 2020.3 | All SITBs | Children/ Adolescents | RSA | Discussion with mother, designed to provoke emotional arousal and disagreement | 0.0424 | 0.0667 | -0.4637 | 0.5486 |
| Kaufman, 2020.4 | All SITBs | Children/ Adolescents | RSA | Discussion with mother on the early conflict-provoking topics | -0.1592 | 0.0669 | -0.3477 | 0.666 |
| Koenig, 2017a.1 | Non-suicidal self-harm | Children/ Adolescents | HR | Baseline/ Resting-state | 0.3204 | 0.0675 | -0.189 | 0.8298 |
| Koenig, 2017a.2 | Non-suicidal self-harm | Children/ Adolescents | RMSSD | Baseline/ Resting-state | 0.005 | 0.0667 | -0.5011 | 0.5111 |
| Koenig, 2017 b.3 | Non-suicidal self-harm | Children/ Adolescents | RMSSD | Cold Pressor Test | -0.3588 | 0.0677 | -0.1514 | 0.8689 |
| Koenig, 2017b.4 | Non-suicidal self-harm | Children/ Adolescents | HR reactivity | Cold Pressor Test | -0.2333 | 0.0671 | -0.2745 | 0.7411 |
| Wielgus, 2016.1 | All SITBs | Children/ Adolescents | RSA | Baseline/ Resting-state | -0.2643 | 0.0777 | -0.8108 | 0.2822 |
| Wielgus, 2016.2 | All SITBs | Children/ Adolescents | RSA | Cognitive stressor task | -0.1841 | 0.0776 | -0.73 | 0.3618 |
| Wielgus, 2016.3 | All SITBs | Children/ Adolescents | RSA | Recovery to Cognitive stressor task | -0.4846 | 0.0785 | -1.0338 | 0.0646 |

## Figure S1. Funnel plot assessing publication bias for studies investigating the association between cardiac measures and SITBs.

***
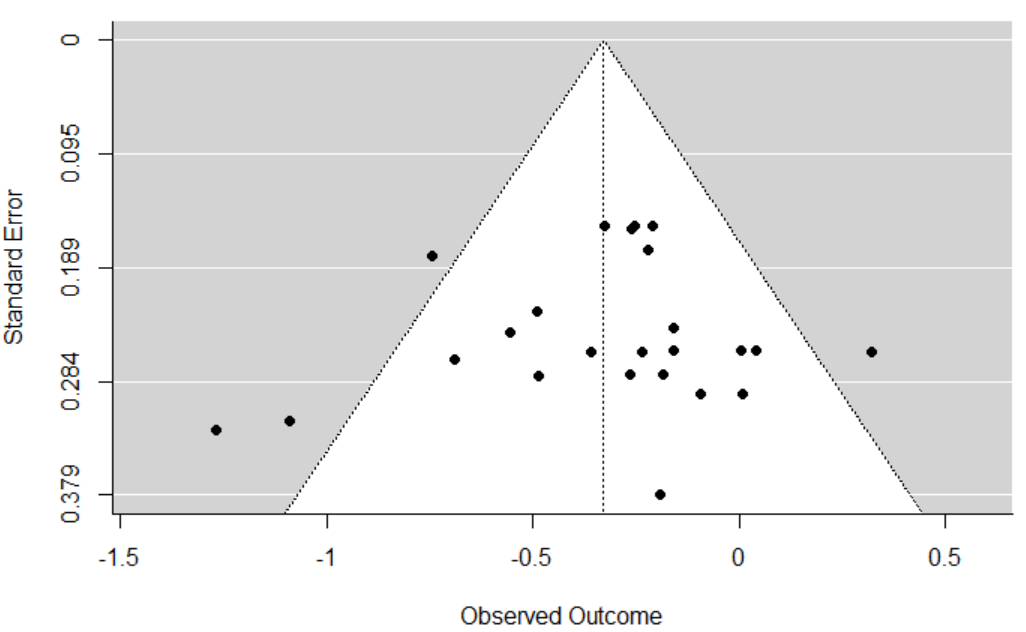
***

# Appendix S7. Meta-analysis on electrodermal measures

## Table S4. Summary of studies investigating the association between electrodermal measures and SITBs

| **Study** | **SITB domain** | **Developmental Stage** | **Electrodermal measure** | **Experimental Task/Activity** | **Hedge's g** | **Variance (g)** | **Lower 95% CI** | **Upper 95% CI** |
| --- | --- | --- | --- | --- | --- | --- | --- | --- |
| Aldrich, 2018.1 | All SITBs | Children/ Adolescents | SCRs | Baseline/ Resting-state | -0.5262 | 0.0742 | -1.06 | 0.0076 |
| Aldrich, 2018.2 | All SITBs | Children/ Adolescents | SCRs | Stress task (anagram solving) | -0.4285 | 0.0737 | -0.9608 | 0.1038 |
| Crowell, 2005.1 | All SITBs | Children/ Adolescents | SCRs | Baseline/ Resting-state | -0.3269 | 0.0882 | -0.9088 | 0.2551 |
| Crowell, 2005.2 | All SITBs | Children/ Adolescents | SCRs | Sadness-evoking video | -0.0497 | 0.087 | -0.6278 | 0.5283 |
| Crowell, 2005.3 | All SITBs | Children/ Adolescents | SCRs | Recovery to Sadness-evoking video | -0.202 | 0.0874 | -0.7815 | 0.3775 |
| Nock, 2008 | Non-suicidal self-harm | Children/ Adolescents | SCRs | Distress Tolerance Test based on Wisconsin Card Sort Test | 0.567 | 0.0512 | 0.1234 | 1.0107 |
| Tatnell, 2018.1 | Non-suicidal self-harm | Young adults | SCL | Baseline/ Resting-state | 0.1047 | 0.0589 | -0.3711 | 0.5805 |
| Tatnell, 2018.2 | Non-suicidal self-harm | Young adults | SCRs | Trier Social Stress Test - Speech task | 0.1981 | 0.0591 | -0.2785 | 0.6747 |
| Tatnell, 2018.3 | Non-suicidal self-harm | Young adults | SCRs | Trier Social Stress Test Exposure to emotion-inducing pictures | 0.201 | 0.0591 | -0.2756 | 0.6776 |
| Tatnell, 2018.4 | Non-suicidal self-harm | Young adults | SCRs | Exposure to emotion-inducing pictures | 0.3259 | 0.0596 | -0.1525 | 0.8042 |
| Tuna, 2021.1 | Non-suicidal self-harm | Young adults | SCL | Baseline/ Resting-state | -0.2058 | 0.0575 | -0.6757 | 0.2642 |
| Tuna, 2021.2 | Non-suicidal self-harm | Young adults | SCRs | Cold Pressor Test | 0.3193 | 0.0579 | -0.1525 | 0.791 |
| Tuna, 2021.3 | Non-suicidal self-harm | Young adults | SCRs | Cold Pressor Test | 0.447 | 0.0586 | -0.0277 | 0.9216 |
| Wang, 2021.1 | Suicide attempt | Young adults | SCRs | Iowa Gambling Task | -0.2308 | 0.1304 | -0.9386 | 0.4771 |
| Wang, 2021.2 | Suicide ideation | Young adults | SCRs | Iowa Gambling Task | -0.0915 | 0.0962 | -0.6994 | 0.5165 |

## Figure S2. Funnel plot assessing publication bias for studies investigating the association between electrodermal activity and SITBs.


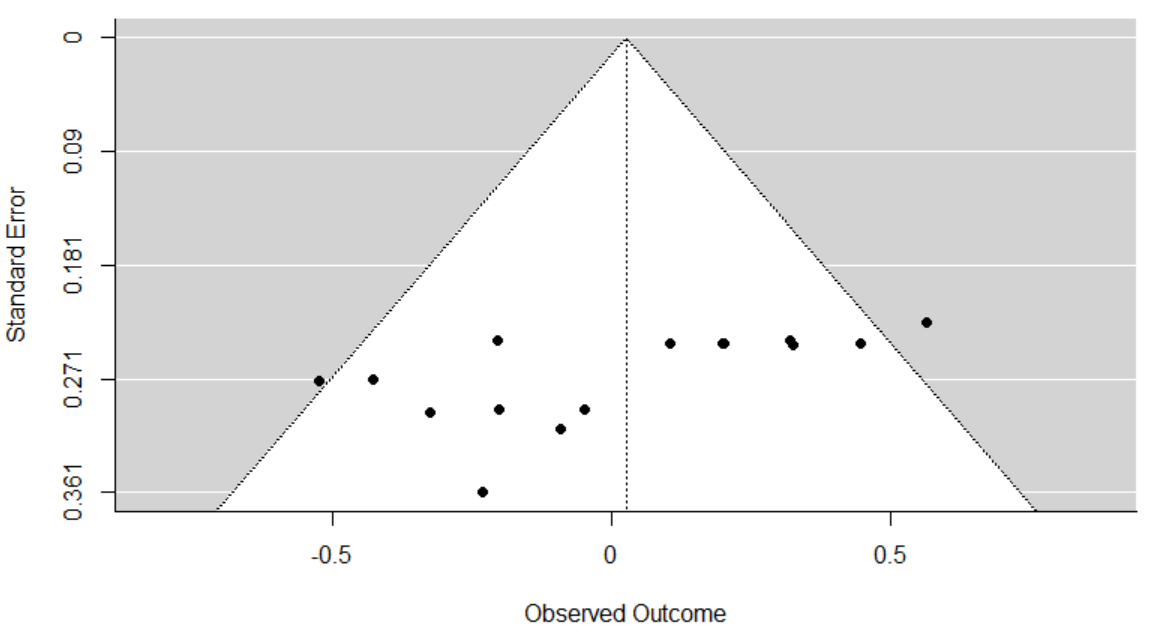

Supplement: Supplementary file 1 — Supporting Information S1 [file JCV2-3-e12148-s001.docx]
